# Lung Surfactant Accelerates Skin Wound Healing: A Translational Study with a Randomized Clinical Phase I Study

**DOI:** 10.1038/s41598-020-59394-5

**Published:** 2020-02-13

**Authors:** Ursula Mirastschijski, Igor Schwab, Vincent Coger, Ulrich Zier, Carmela Rianna, Wei He, Kathrin Maedler, Sørge Kelm, Arlo Radtke, Gazanfer Belge, Patrick Lindner, Frank Stahl, Martin Scharpenberg, Lukas Lasota, Jürgen Timm

**Affiliations:** 1 Center for Biomolecular Interactions Bremen, Faculty of Biology and Chemistry, University of Bremen, Bremen, Germany; 2 Department of Plastic, Reconstructive and Aesthetic Surgery, Klinikum Bremen-Mitte, Bremen, Germany; 3 Department of Experimental Plastic Surgery, Kerstin Reimers Laboratory for Regeneration Biology, Hannover Medical School, Hannover, Germany; 4 Institute of Biophysics, University of Bremen, Bremen, Germany; 5 Faculty of Biology and Chemistry, University of Bremen, Bremen, Germany; 6 Institute of Technical Chemistry, Leibniz University Hannover, Hannover, Germany; 7 University of Bremen, Competence Center for Clinical Trials Bremen, Bremen, Germany

**Keywords:** Drug development, Preclinical research, Translational research

## Abstract

Lung surfactants are used for reducing alveolar surface tension in preterm infants to ease breathing. Phospholipid films with surfactant proteins regulate the activity of alveolar macrophages and reduce inflammation. Aberrant skin wound healing is characterized by persistent inflammation. The aim of the study was to investigate if lung surfactant can promote wound healing. Preclinical wound models, e.g. cell scratch assays and full-thickness excisional wounds in mice, and a randomized, phase I clinical trial in healthy human volunteers using a suction blister model were used to study the effect of the commercially available bovine lung surfactant on skin wound repair. Lung surfactant increased migration of keratinocytes in a concentration-dependent manner with no effect on fibroblasts. Significantly reduced expression levels were found for pro-inflammatory and pro-fibrotic genes in murine wounds. Because of these beneficial effects in preclinical experiments, a clinical phase I study was initiated to monitor safety and tolerability of surfactant when applied topically onto human wounds and normal skin. No adverse effects were observed. Subepidermal wounds healed significantly faster with surfactant compared to control. Our study provides lung surfactant as a strong candidate for innovative treatment of chronic skin wounds and as additive for treatment of burn wounds to reduce inflammation and prevent excessive scarring.

## Introduction

Inappropriate skin wound healing is marked by either chronic, non-healing ulcers or excessive scarring with a sustained inflammatory reaction. The current state-of-the-art treatment is surgical removal of diseased tissues followed by plastic-reconstructive wound closure or long-term treatment with various wound dressings. The social impact of aberrant wound repair manifests in severely reduced patient’s life quality, absence from work and high health care costs with up to 18 million GBP/25 billion USD per year for chronic wounds^1,2^ or 0.3 billion EUR^3^/12 billion USD^2^ for scarring. Bioactive substances for wound treatment were tested for decades without clinical success^4^. With regard to excessive scarring, no preventive treatment is currently available^5^.

The inflammatory phase during skin wound healing is an important step towards wound closure. Loco-regional cells are activated by pro-inflammatory mediators, epithelial cells resurface the wound bed and fibroblasts produce new matrix for defect replacement^6^. The innate immune system is activated, neutrophils and monocytes invade the wound bed to clear debris and microbes^7^. During this process they release factors critical for wound closure. Increased inflammation delays wound closure and is thought to be the underlying cause for both, chronic non-healing wounds and excessive scarring^6^.

In the lung, type II alveolar epithelial cells produce surfactant that covers as a surface film the air-liquid-interphase between the lung parenchyma and the air^8^. Pulmonary surfactant is mainly constituted of phospholipids (80%), cholesterol (10%), the hydrophilic surfactant proteins (SP)-A and D and the lipophilic proteins SP-B and SP-C (2–5%)^9,10^. The phospholipids can be categorized into phosphatidylcholine (80%), phosphatidylethanolamine and the anionic phosphatidylglycerol (10%)^10^. Lung surfactant is highly important for intrapulmonal biomechanics, e.g. it stabilizes alveoli and cells upon mechanical strain or high surface tension, prevents the alveolar collapse at the end of the expiration and edema formation^9^.

Lung surfactants are used as standard therapy for reducing alveolar surface tension in preterm infants^11,12^. Phospholipid films with surfactant proteins regulate the shape and activity of alveolar macrophages by controlling surface tension^13^, by binding and opsonizing pathogens^14^ or by its own antimicrobial activity^15^. For treatment of respiratory distress syndrome, lung surfactants of bovine or porcine origin are commercially available, approved by the respective authorities, tested and licenced for pulmonary application. The natural lung surfactant lipoprotein complex consists of lipids, predominantly dipalmitoylphosphatidylcholine (DPPC), and four surfactant proteins (SP), e.g. the hydrophilic SP-A and SP-D and the lipophilic SP-B and SP-C^13^. Commercially available lung surfactants lack SP-A and SP-D due to the harvesting process of the lipophilic components^16^. Around 90% of the surfactants are lipids with the major compound DPPC. Interestingly, phospholipid fractions can bind and subsequently block Toll-like receptor-4 interacting proteins CD14 and MD-2 resulting in anti-inflammatory properties of lung surfactants^17^. For example, pre-incubation of human monocytic cells with DPPC attenuated the leukocyte inflammatory response via downregulation of protein kinase C^18^. Furthermore, lung surfactant preparations block IκB kinase, ERK and p38 MAP kinase activity and thus inhibit pro-inflammatory signals^19^.

Based on these anti-inflammatory properties of surfactants in the lung, we hypothesized that lung surfactants will promote healing of chronic skin wounds and ameliorate scarring. Similar to the lung, the skin is exposed to the air-liquid-interphase, it consists of epithelial and mesenchymal cells and reacts with fibrosis to chronic inflammatory repair processes^6^. The aim of this study was to modulate and reduce inflammation in skin wounds by topical treatment with lung surfactant, to enhance cutaneous wound healing and, ultimately, to reduce excessive scarring. By applying *in vitro* and *in vivo* experimental models followed by a human clinical phase I study, we demonstrate that topically applied lung surfactant is suitable for skin treatment, reduces local inflammation and accelerates human wound closure.

## Results

Different experimental wound models were used to study keratinocyte migration and the inflammatory reaction to a wounding stimulus *in vitro* and *in vivo*. Encouraging results of pre-clinical experiments led to a clinical phase 1 study on human volunteers. The bovine lung surfactant Alveofact^®^ (Alv) was diluted in saline and applied at different concentrations to cells and wounds. We compared the effect of Alv with groups that were treated with the vehicle saline, which served as control and to fatty gauze, the current clinical standard treatment for open skin wounds.

### The influence of Alveofact treatment on cellular scratch closure *in vitro* and wound healing *in vivo*

**Figure 1:**
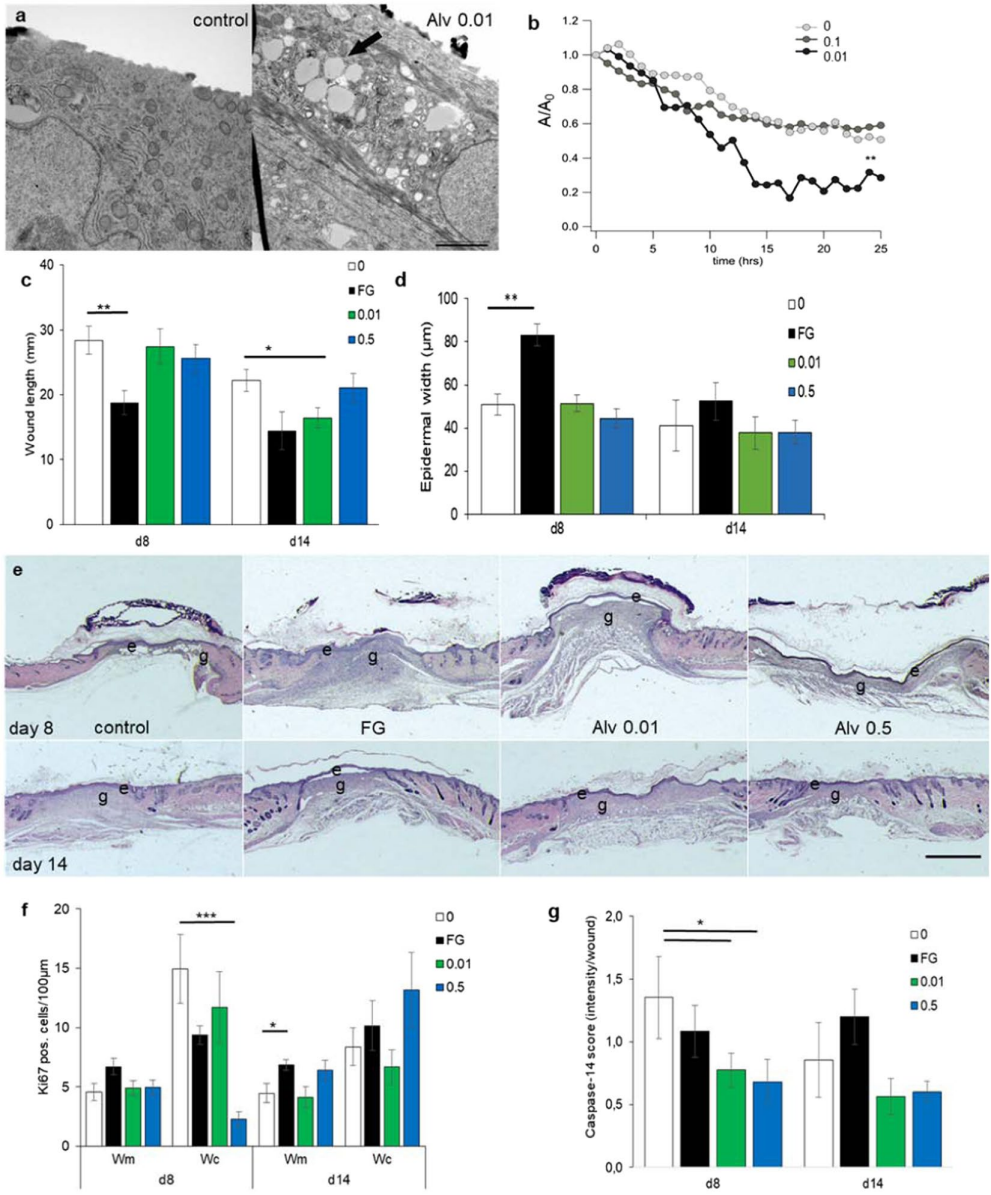
Alveofact accelerates epithelial migration. (**a**) Transmission electron microscopy imaging of lipid micelle uptake into keratinocytes with Alv. Left control, right Alv at 0.01 mg/mL. Paranuclear micelle deposition (arrow); scale bar 2 µm. (**b**) Keratinocytes migrated significantly faster (**p < 0.005) over a scratch with Alv 0.01 compared to control (0) or Alv 0.5 captured over 24 h. A/A_0_ Ratio between final and initial scratch area. (See also Suppl videos). (**c**,**d**) Wound length (distance between normal dermis margins in µm; c) and epidermal thickness (in µm; d) were assessed by HE-stained sections. (**c**) Fatty gauze treatment decreased wound length significantly faster on d8 (**p = 0.006) and Alv 0.01 on d14 (*p = 0.01) com*p*ared to control. (**d**) Statistically significant thickening was noticed with fatty gauze compared to control. (**e**) The neo-epidermis of control, Alv 0.01 or Alv 0.5 was thin with fluffy granulation tissue in contrast to epidermal thickening with compact dermal tissue with fatty gauze on d8. HE section, labeling for upper and lower panels: e epidermis, g granulation tissue; 0 control, FG fatty gauze (clinical standard), 0.01 mg/mL Alv and 0.5 mg/mL Alv. Scale bar 2 mm. (**f-g**) Keratinocyte proliferation was assessed by Ki67 (**f**) and epidermal differentiation by caspase-14 (**g**) immunohistochemistry in wound margins (600 µm) and centres (500 µm). (**f**) Significantly more Ki67-positive cells were found per 100 µm epithelial length in wound centers of controls compared with Alv 0.5 on d8 (*p* = 0.001) and more in FG on d14 (*p* = 0.03). (**g**) Significantly less apoptotic cells (expressed as intensity per wound) were found with Alv 0.01 and Alv 0.5 compared to control on d8 (*p* = 0.04). *p < 0.05; ***p < 0.005. Bars in white 0 control; black FG fatty gauze; green 0.01 Alv; blue 0.5 Alv. d8: n = 6; d14 n = 5 (control, FG), n = 6 (Alv 0.01, Alv 0.5) animals per group. wm wound margin, wc wound center. Student’s t-test. Mean ± SEM.

In an *in vitro* scratch model, keratinocytes migrate through the scratch to close the gap between the cells. Electron microscopy imaging showed uptake of Alv micelles with perinuclear storage in keratinocytes (Fig. 1a right panel) whereas control-treated keratinocytes were devoid of such micelles (Fig. 1a left panel). In this cell culture system, Alv at 1 mg/mL inhibited significantly cell migration over a scratched gap compared to vehicle control for both fibroblasts and keratinocytes at 12 and 24 h after treatment (*p* < 0.001, Suppl. Fig. 1a,b), whereas at lower concentrations (0.1 or 0.01 mg/mL) Alv did not affect fibroblast migration (Suppl. Fig. 1b). Alv had only minor influence on fibroblast contractility in free-floating collagen gels with slightly less gel contraction at 0.1 and 1.0 mg/mL (Suppl. Fig. 1c). In contrast to fibroblasts, keratinocyte migration was accelerated by incubation with 0.01 mg/mL in comparison to control with a 2.6 fold reduction of the denuded scratch area in controls, 3.6 fold with Alv 0.01 and 1.8 fold with Alv 0.1 after 12 h incubation (Suppl. Fig. 1a). In a separate experiment, cellular migration behaviour over a scratch was monitored using live-imaging over 25 h. Keratinocytes treated with 0.01 mg/mL Alv migrated significantly faster than control-treated cells (*p* < 0.005; Fig. 1b; video in Suppl. Video 1– 3).

In order to investigate whether this effect of Alv observed *in vitro* leads to consequences *in vivo*, a standardized animal wound healing model was applied. Full-thickness excisional wounds in mice were treated topically with Alv at 0.01 or 0.5 mg/mL, with saline as control or fatty gauze as current clinical standard treatment for acute wounds. With regard to macroscopic wound closure rates, no differences were found between groups (data not shown). Since mice heal more by wound contraction than reepithelialization^20,21^, reduced wound length implicates faster wound contraction, whereas increased epidermal thickness indicates enhanced epithelial proliferation. Histologically, increased wound area reduction and shortest epidermal wound length were observed with fatty gauze on d8 (*p* < 0.05) whereas results were similar for control, 0.01 or 0.5 mg/mL Alv treated animals (Fig. 1c). On d14, the wound length was shorter with Alv 0.01 compared to saline and similar to fatty gauze (Fig. 1c). With fatty gauze treatment, epidermal thickness almost doubled in comparison to control on d8 (Fig. 1d; *p* = 0.006) and had the histological appearance of a hypertrophic scar with cell and collagen rich dermal layers (Fig. 1e). This was reduced after 14 days, but still 1.4 times thicker than the epidermal width seen with Alv treatment. With control and Alv treatment, thin epidermal layers and also a fluffy dermal compartment were noticed without any hint to excessive scarring (Fig. 1e). Hence, faster wound contraction seen with fatty gauze appears to be at the cost of epidermal hypertrophy and thickening.

**Figure 2:**
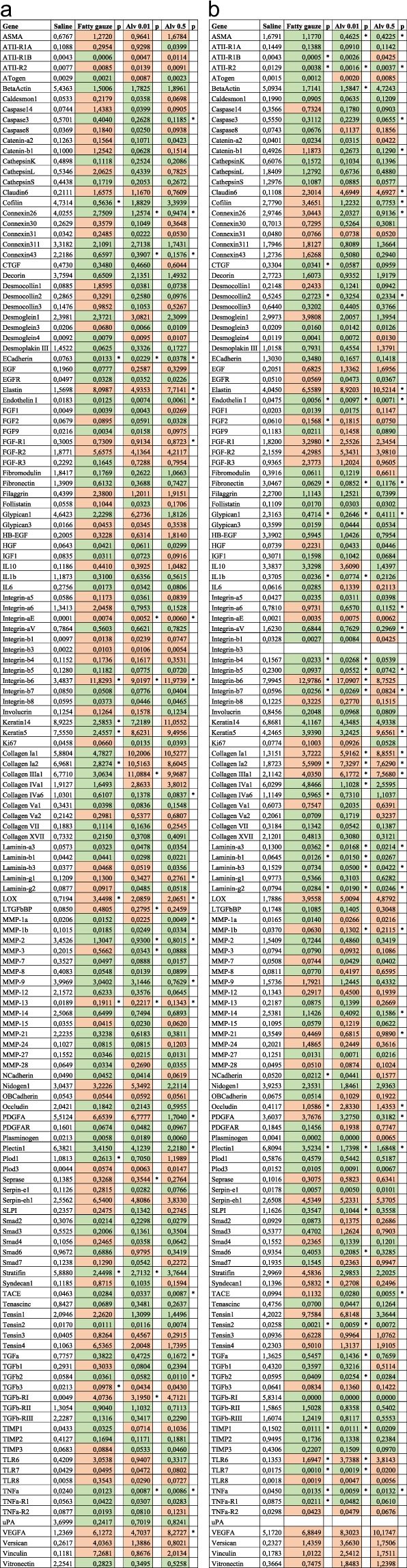
Gene array analysis of day 8 and 14. Complete data set of the gene array analysis of wound tissues excised at day 8 (**a**) and day 14 (**b**). Upregulated genes with Alv 0.01 or 0.5 mg/mL or Fatty gauze treatment in comparison with saline are marked in red, downregulated in green. Statistically significant values (p<0.05) are marked in an asterisk* to the right of each column.

Enhanced cellular migration observed in cell culture experiments was reflected by elevated expression of pro-migratory genes in homogenized skin wounds analyzed by a wound tissue microarray (Fig. 2). Pro-migratory *MMP-13* and *integrin-β6* expression were significantly increased with Alv treatment at d8 (*p* < 0.05; Fig. 2a).

Epithelial proliferation and apoptosis were assessed by Ki67 or caspase-14 immunohistochemistry, respectively. At d8, keratinocyte proliferation was highest in the wound centre of untreated control mice and significantly reduced by Alv 0.5 (p < 0.005; Fig. 1f). No differences in proliferation were found in wound centres at d14. Differentiating epithelial cells showed significantly reduced apoptosis with Alv 0.01 and 0.5 compared to control on d8 and compared to the fatty gauze on d14 by caspase-14 immunohistochemistry (Fig. 1g) and gene array analysis (Suppl. Fig. 1d).

### Alveofact attenuates wound inflammation

**Figure 3:**
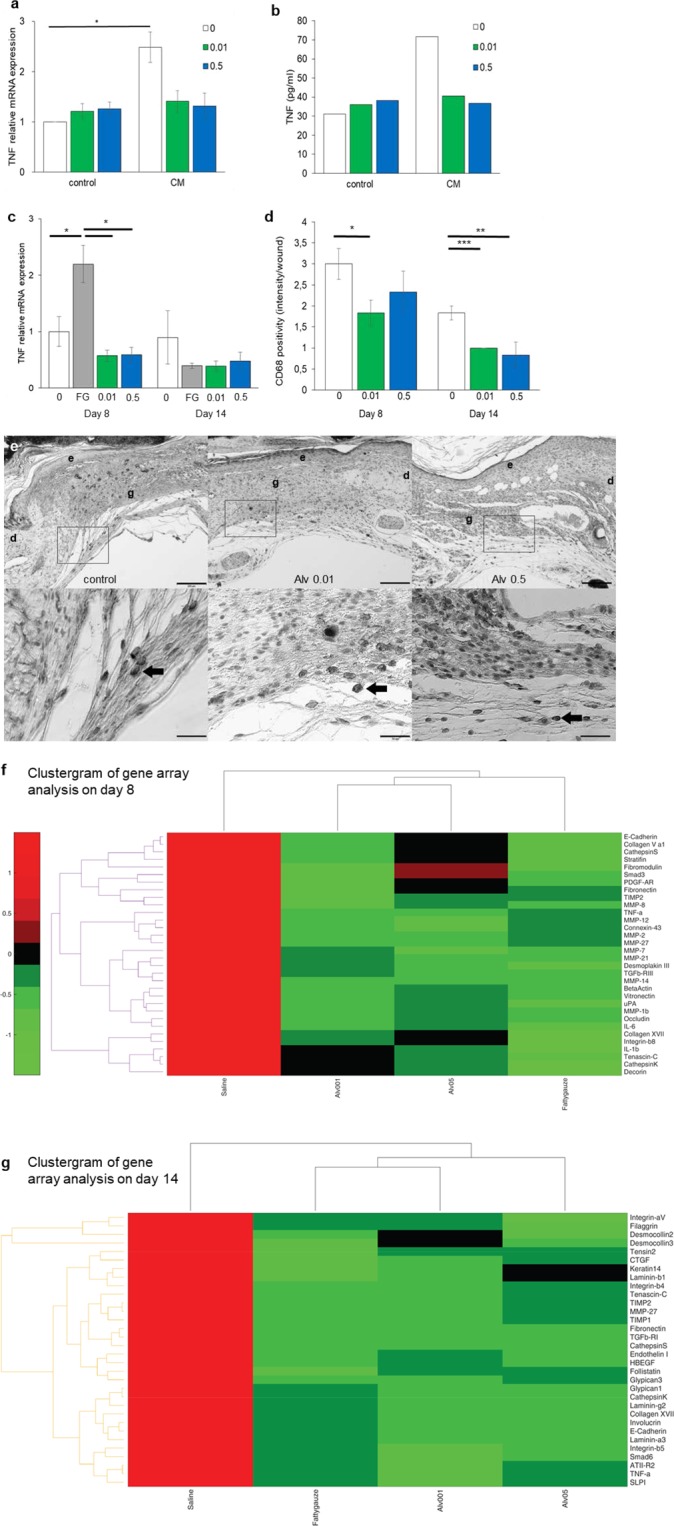
Alveofact attenuates wound inflammation. (**a,b**) *TNF* mRNA (**a**) and protein (**b**) expression in PBMCs and media. (**a**) *TNF* mRNA was significantly (*p<0.05) increased in conditioned media (CM) compared to fresh media (control) quantified by qRT-PCR. This effect was not seen with Alv. n = 3. (*p<0.05). (**b**) TNF protein was increased in conditioned PBMC media without treatment compared to fresh media. No increase was noted with Alv. n = 1. (**c**) TNF mRNA expression in wounds was determined by qRT-PCR. Significantly more *TNF* expression was found with fatty gauze compared to control or both Alv concentrations on d8. *p<0.05, **p<0.01. n = 5–6. Mean ± SEM (Student’s t-test). (**d,e**) Macrophage infiltration was assessed in full-thickness excisional wound tissue by CD68 immunohistochemistry (n = 12 per group). (**d**) Significantly less macrophage infiltration was found with Alv 0.01 (d8 + d14) and Alv 0.5 (d14) compared to control. CD68 intensity was assessed by positive cell count per mm^2^ area. White bars 0 control; grey bars fatty gauze; green bars Alv 0.01; blue bars Alv 0.5 (Student’s t-test; *p = 0.05, **p = 0.007, ***p = 0.0003, n = 12 per group). (**e**) Tissue sections from control, Alv 0.01 or Alv 0.5 of d8 (CD68 dark staining, indicated by arrows). Upper lane scale bar 200 µm, close-ups in lower lane scale bar 50 µm. e epidermis, d dermis, g granulation tissue. (**f,g**) Clustergrams of gene array showing up- and downregulation of the pro-inflammatory genes *TNF, IL-1β* and *IL-6* in comparison to control and the clinical standard fatty gauze (FG). (**f**) Gene regulation on day 8. (**g**) Gene regulation on day 14. Data are presented with a standard red-green-map in which red represents values above the mean, black represents the mean, and green represents values below the mean of a row (gene) across all columns (samples). 0 control, FG fatty gauze, Alv at 0.01 and 0.5 mg/mL.

To investigate whether Alv influences the interaction between keratinocytes and immune cells, primary human keratinocyte cultures were wounded by a scratch and then incubated with or without Alv for 24 h. Culture media were collected and added to human peripheral blood monocytic cells (PBMC) cultures. TNF mRNA (Fig. 3a) and protein (Fig. 3b) expression were increased by the 2.5-fold or 2.3-fold, respectively, when PBMCs were incubated with conditioned media from keratinocyte cultures, indicating a paracrine pro-inflammatory effect from damaged keratinocytes. In contrast, supernatants from Alv-treated keratinocytes did not induce such a pro-inflammatory reaction in PBMCs (Fig. 3a,b) nor did Alv itself have such an effect (control media, no pre-conditioning).

In wounds, reduced *TNF* gene expression (Figs. 2 and 3) and mRNA (Fig. 3c) were found with Alv in comparison to control on days 8 and 14. Significant differences were noted between control and Alv at both concentrations at days 8 and 14 by gene array analysis (Fig. 2). In agreement with the array data, significantly increased expression of *TNF* mRNA was found using qRT-PCR with fatty gauze treatment at d8 in comparison to control (*p* < 0.05) (Fig. 3c). At both concentrations applied, Alv-treated skin showed markedly reduced *TNF* expression (both *p* < 0.01) compared to fatty gauze at d8 (Fig. 3c), which mirrored the gene array results. No significant differences were found for Alv in comparison with controls.

*In vivo*, macrophages dominate wound healing during the inflammatory phase between days 3 and 10 and stimulate inflammatory processes by secretion of various cytokines and growth factors^22^. Macrophages were counted in sections of wounds stained with CD68 immunohistochemistry and expressed as positive cells per mm^2^ skin tissue (Fig. 3d,e). Significantly less CD68 positive cells were found in Alv treated skin wounds (n = 12 per group) compared to controls. Alv 0.01 reduced CD68 positivity 2-fold on d8 (*p* = 0.05) and 4-fold on d14 (*p* = 0.0003) and Alv 0.5 reduced it 2.6-fold on day 14 (*p* = 0.007) compared to controls (Fig. 3d). Macrophages are the predominant source of pro-inflammatory TNF in wounds. Significantly reduced pro-inflammatory *TNF* was found with Alv 0.01 and Alv 0.5 by gene array analysis (Fig. 3f,g) in skin wounds at d8 (*p* ≤ 0.01) and d14 (*p* ≤ 0.01, Fig. 2). Alv 0.5 reduced *TACE* expression significantly during the whole observation period (d8: *p* < 0.001; d14: *p* < 0.05, Fig. 2). Furthermore, Alv 0.01 treatment reduced *IL-1β* expression significantly (*p* < 0.01) at d14 with a similar trend seen for *IL-6* (Fig. 2).

### Alveofact influenced tissue remodelling

**Figure 4:**
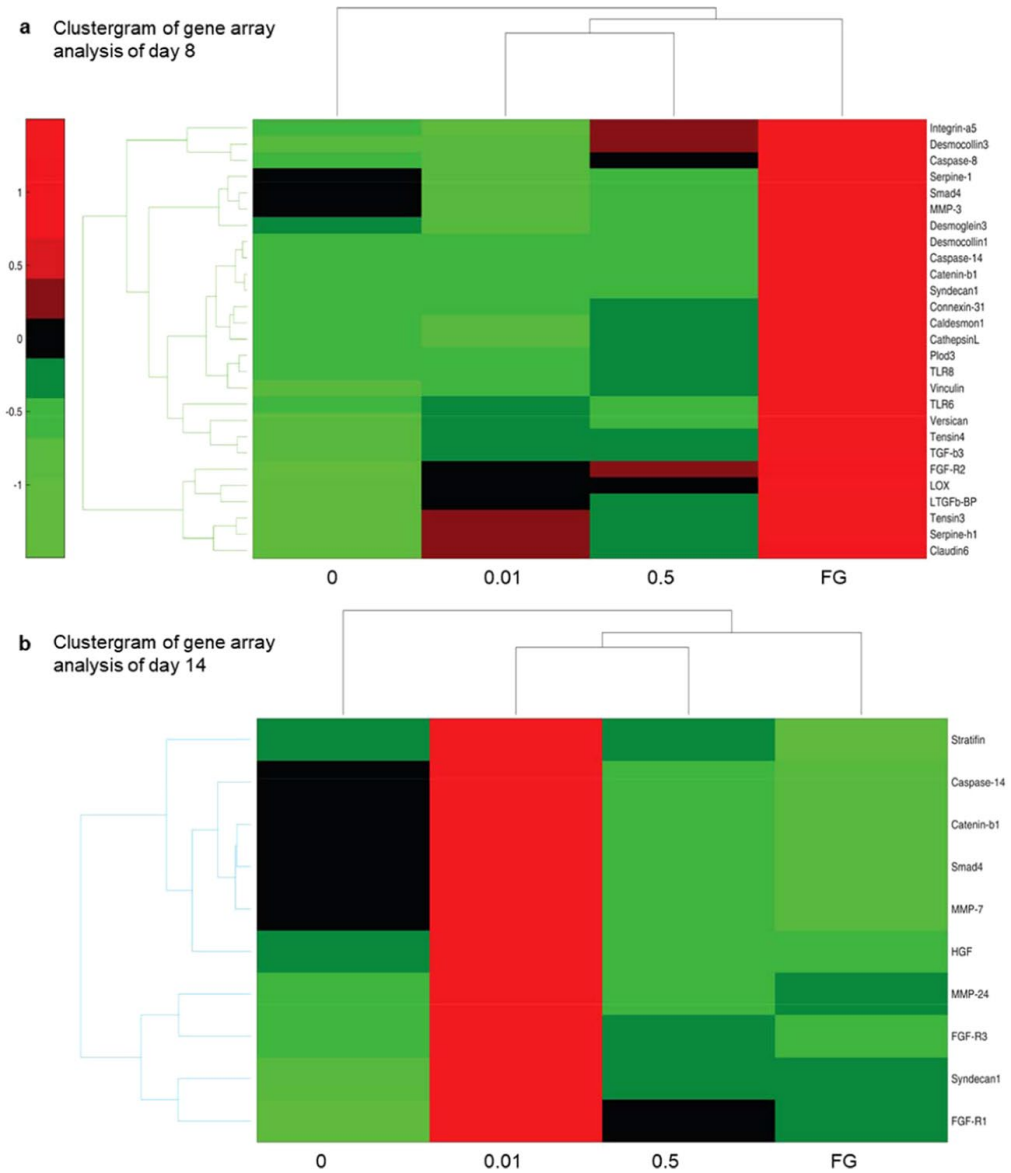
Clustergram of gene array showing up- and downregulation of genes for tissue remodelling and scarring by Alveofact in comparison to control (saline) or fatty gauze. (**a**) Gene regulation on day 8. (**b**) Gene regulation on day 14. Note reduced *MMP-3* expression on d8 and reduced *β-catenin* on both days reflecting measurements for *E-cadherin* expression (see Fig. 3). Data are presented with a standard red-green-map in which red represents values above the mean, black represents the mean, and green represents values below the mean of a row (gene) across all columns (samples). 0 control, FG fatty gauze, Alv at 0.01 and 0.5 mg/mL.

MMP-3 is a pro-inflammatory and pro-fibrotic MMP that cleaves E-cadherin and facilitates cellular migration by disengaging intercellular bonds^23^. Downstream signalling of MMP-3 after E-cadherin cleavage leads to the release of intracellular β-catenin with subsequent translocation into the nucleus for transcriptional activation of genes important for tissue remodelling and fibrosis. *MMP-3* expression was significantly (*p* = 0.034) reduced in samples with Alv 0.01-treated wounds compared to control on d8 (Fig. 2a, Fig. 4a) with a concomitant significant reduction of *E-cadherin* expression and a trend to significance on d14 (Fig. 2b). *β-catenin* (Fig. 4a,b) gene expression was reduced with Alv 0.01 on d8 (Fig. 2a) and for both Alv concentrations on d14 compared to control (Fig. 2b).

Wound contraction is part of skin wound healing and reduces the wound surface. Transforming growth factor-(TGF)-β initiates the differentiation of fibroblasts into highly contractile myofibroblasts that contribute to wound contraction. Downregulation of profibrotic *TGF-β2, TGFβ-RI* and *MMP-3* were found at d8 and, in part, at d14 by gene array analysis (Fig. 2).

**Figure 5:**
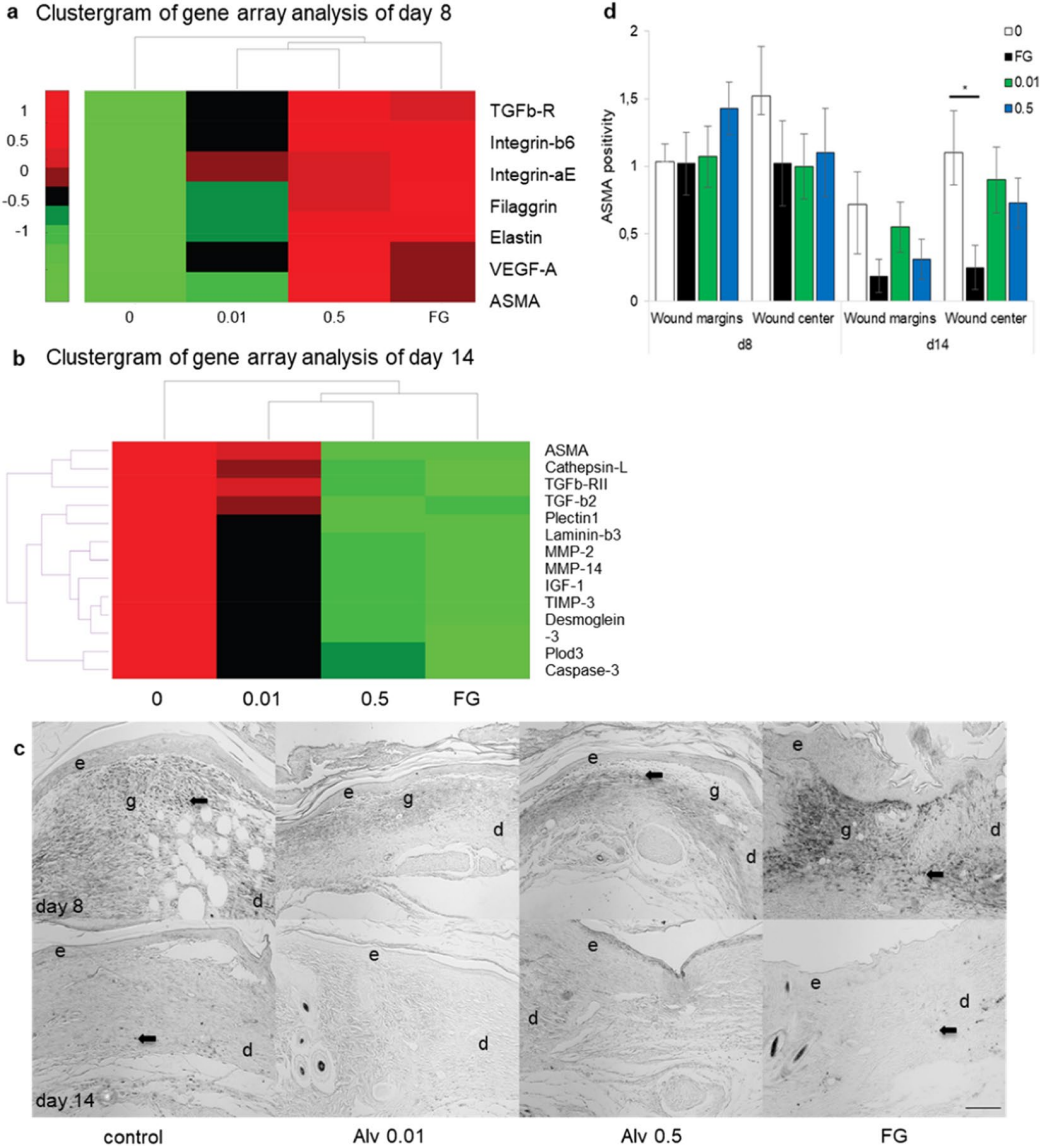
Lung surfactant decreases ASMA expression in wounds on day 14 (**a,b**) Alph-smooth-muscle actin (ASMA) analysis by gene array. An increase of *ASMA* expression was found in all groups compared to control on d8 (**a**). Significantly reduced *ASMA* was found with both Alv treatments on d14 (b, see also Fig. 2). (**c,d**) ASMA positivity in wounds (arrows) was assessed by scoring 0–3 of immunohistochemical sections. Wound margins and centres had a trend to less ASMA with Alv compared to controls on d14 (**c**,**d**) with significantly less ASMA in FG (*p = 0.048). (**e**) epidermis, d dermis, g granulation tissue, FG fatty gauze. Scale bar in d14 for all sections: 200 µm. (**d**) White bars 0 control, green bars 0.01 Alv, blue bars 0.5 mg/ml Alv, black bars fatty gauze. d8: n = 12 all groups, d14: n = 12 control, Alv 0.5, n = 10 Alv 0.01, n = 8 FG. Mean ± SEM.

α-Smooth-muscle actin (ASMA) is a marker for the myofibroblasts. By gene array analysis, an increase in *ASMA* expression was found in all groups in comparison with control on d8 (Figs. 2a and 5a). On d14, this trend changed with significantly less *ASMA* in both Alv groups compared to control (*p* < 0.05; Figs. 2b and 5b). Furthermore, significantly (*p* < 0.05) reduced *AngiotensinII-Receptor-2 (ATII-R2*) was found with Alv in comparison with control (Fig. 2). Notably, pro-fibrotic connective tissue growth factor (*CTGF/CCN2*) was reduced with both Alv concentrations at d14 (Fig. 2). ASMA immunohistochemistry (Fig. 5c,d) showed no significant differences (ANOVA test for multiple comparisons) between control and Alv 0.01 or 0.5 at both time points. On d14, fatty gauze had the lowest ASMA values, an indicator for accomplished wound closure and disappearance of contractile myofibroblasts (Fig. 5d).

To summarize preclinical results, Alv had beneficial effects on skin wound healing presumably by enhancing epithelial migration and reducing pro-inflammatory cytokines *in vitro* and *in vivo*. As a consequence, the next step was a translational study to investigate the effect of Alv on human skin wound healing *in vivo*.

### Alveofact was safe and well tolerated when applied topically onto intact and wounded human skin: results of a randomized, prospective clinical phase I study

**Figure 6:**
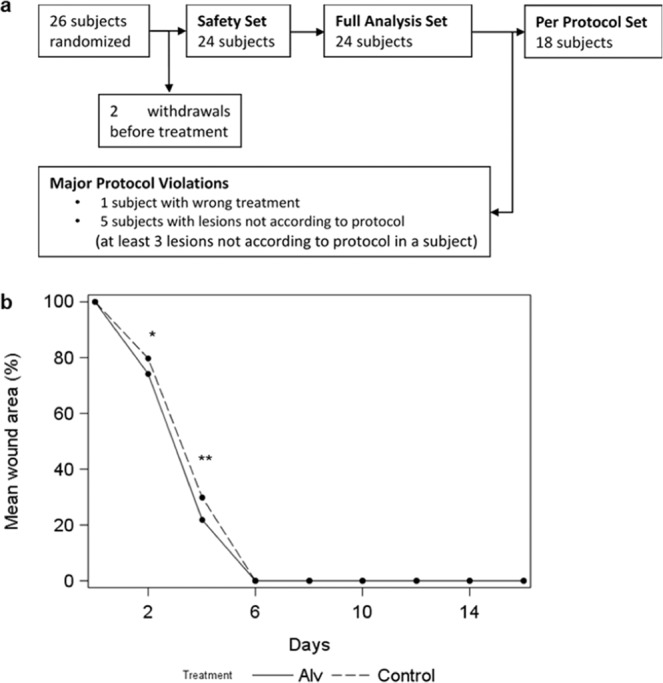
Topical lung surfactant accelerates significantly skin wound closure of human suction blister wounds *in vivo*. (**a**) Subject number flow chart according to CONSORT guidelines. Twenty-six subjects participated in the study, 2 withdrew before initiation of study treatment. Six subjects were excluded from the per protocol (PP) set due to major protocol violations, resulting in 18 subjects that were analysed in the PP set. (**b**) Progression of %-mean-wound-area over time of observation. At d2 and 4, Alv treated wounds presented with significantly smaller %-mean-wound area than controls indicating faster healing in the Alv arm. Total wound closure (0%-mean-wound-area) was achieved at d6 for all wounds. n = 24, FAS cohort. Continuous line Alv, dotted line, control. *p = 0.02; **p = 0.003 (Wilcoxon Signed Rank Test).

Primary endpoints of this prospective, randomized, translational clinical phase I study were the safety and tolerability of surfactant when applied onto normal skin. The application of Alv on intact skin was assessed by a standardized clinical scoring scale (CSS). Secondary endpoints were wound closure time and pain related to treatment of superficial skin wounds. We used a human suction blister wound model^24^ to investigate potential beneficial effects of lung surfactant on skin wound healing. The analysis of the primary and secondary target variables was performed on the per-protocol set (PP) or the full-analysis set (FAS) according to CONSORT guidelines (Fig. 6a).

#### Demographics and baseline characteristics

The actual study collective consisted of healthy female (15) and male (9) participants of a mean age of 25.6 years, mean Body-Mass-Index (BMI) of 24.2. Volunteers were recruited and experiments performed within a period of 10 months between December 2016 and September 2017. Summary statistics of vital parameters like systolic/diastolic blood pressure (in mm Hg) and pulse (bpm) were within normal limiting values (Table 1). For the analysis of the clinical phase I study, one subject was excluded from PP, because the administered treatment differed from the randomized treatment, 5 subjects were excluded from PP because the lesions were not according to protocol (at least 3 lesions not according to protocol) which was due to air-leakage of the suction blister device. Consequently, the final analysed PP consisted of 18 patients as shown in the flow diagram according to CONSORT guidelines^25^ (Fig. 6a). In summary, twenty-six subjects participated in the study, from which 2 withdrew before initiation of study treatment. No blister lesions were produced for those subjects and no data was collected. Consequently, they were replaced (two additional subjects were randomized), resulting in a total number of 26 randomized subjects, to achieve 24 evaluable subjects.

#### Primary target variable CSS (safety parameter)

Mean CSS values were similar between Alv and control (saline) treated arms with slightly better values in the Alv group (difference Alv – control = −0.02, Table 2). From the clinical aspect, wound erythema impressed less with Alv compared to control. Based on the CSS values the Alv treatment is therefore regarded as being comparably safe as the control treatment. Sensitivity analyses with the FAS were in line with PP results (data not shown). The primary endpoint CSS was additionally analysed using treatment and sex as well as their interaction as explanatory variables in an ANCOVA model. No significant differences were found between treatment effects for the different sexes (p-value = 0.4170). Results were confirmed by analysis of data by a mixed model with the same covariates and a random intercept for each subject was computed to take possible correlations of measurements obtained from the same individual into account (*p*-value = 0.2839).

**Table 1:** Summary statistics of metric baseline variables of participants included into the study.

Variable	N	Mean ± SD	Min	Q1	Median	Q3	Max
Age	24	25.58 ± 8.10	18.00	20.00	24.00	27.00	48.00
Weight (kg)	24	70.00 ± 13.26	50.00	60.00	65.00	80.00	97.00
Height (cm)	24	169.70 ± 9.13	152.00	163.50	170.00	179.00	184.00
BMI	24	24.22 ± 3.74	19.38	21.65	23.32	25.33	32.05
Pulse (bpm)	24	76.88 ± 12.13	50.00	68.50	80.00	84.50	97.00
Blood pressure systolic (mmHg)	24	121.30 ± 12.77	101.00	111.00	120.00	128.00	159.00
Blood pressure diastolic (mmHg)	24	77.67 ± 8.54	66.00	70.50	78.00	80.50	97.00

#### Secondary target variable NRS (numerical rating scale; safety parameter)

The mean difference of NRS over all visits between Alv and control was exactly 0 within the PP-population (Table 2). Hence, both treatments did not cause substantial pain on topical skin wounds (highest value for NRS was 1). A sensitivity analysis with the FAS was in line with PP results (data not shown). Although this analysis was of exploratory nature, its results indicate evidence for non-existence of safety concerns with respect to Alv treatment compared to control.

#### Secondary target variable percent-mean-wound-area (efficacy parameter)

The mean differences of percent-mean-wound-area (Alv versus control) at d2 and 4 were negative, indicating a faster wound healing for Alv treated arms. Significant results were found between the percent mean wound area of Alv and control treated arms at both visits (d2: *p* = 0.038 and d4: *p* = 0.003) within the FAS population (n = 24, Fig. 6b, Table 3). At all other visits, there was no difference in wound size between treatment groups. At day 6, the majority of wounds was completely closed. A sensitivity analysis with PP-population was in line with FAS results with slightly lower *p*-values (data not shown). Because sex hormones play an important role in skin wound repair^26^, the wound size (measured in % of initial wound size) was analysed using treatment and sex as well as their interaction as explanatory variables in an ANCOVA model for each visit separately. Wounds were found to be significantly smaller in women at d4 (*p* = 0.0383), indicating faster wound healing. This effect was no longer detectable at d6 (*p* = 0.8069).

**Table 2:** Scoring for skin appearance by the clinical scoring scale (CSS) and for pain using the numerical rating scale (NRS).

Variable	Alv (Mean ± SD)	Control (Mean ± SD)	Alv-Control (Mean ± SD)	p-value
CSS	0.18 ± 0.05	0.20 ± 0.07	−0.02 ± 0.07	<0.0001
Pain (NRS)	0.09 ± 0.09	0.09 ± 0.09	0.00 ± 0.04	<0.0001

The Wilcoxon signed rank test for non-inferiority of Alv compared to control was significant (*p* < 0.0001), indicating that mean CSS and NRS values in Alv treated arms were not worse by more than one score point compared to control treated arms. Mean ± SD, PP collective, n = 18.

#### Secondary target variable TEWL (efficacy parameter)

**Figure 7:**
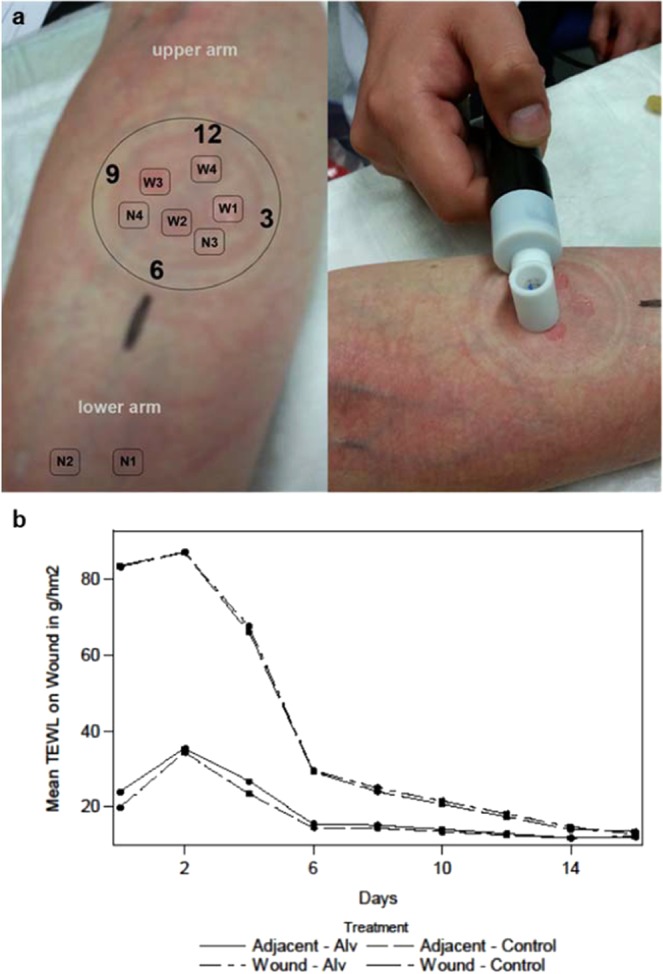
TEWL measurements of intact skin and superficial skin wounds. (**a**) With a suction blister device, a vacuum of 200 mm Hg was applied to the volar side of the lower arm to form blisters. TEWL measurements (right panel) were performed on non-dressed normal skin (N1 + N2), on normal skin covered by the occlusive wound dressing (N3 + N4) and on wounds. Wounds were measured clockwise starting from 3 o’clock (W1) up to 12 o’clock (W4). (**b**) The progression of mean TEWL of wound points. The wound points (W1–4) started with a high mean TEWL level compared to normal (N1 + 2) and adjacent skin points (N3 + 4). Wound levels increased from d0 to d2 and decreased very fast until d6 (dressing period) which indicates a fast healing process with both treatments. TEWL values were similar between treatments. n = 24, FAS cohort. Continuous line Alv, dotted line, control (Wilcoxon Signed Rank Test).

Wound closure was assessed objectively by measuring the transepidermal water loss (TEWL). Mean TEWL values at healthy skin points remained nearly at the same level (with slightly lower values for Alv treated arms) over the entire time of observation (Fig. 7). For adjacent skin points the mean TEWL values increased from d0 compared to d4 and decreased to the level of normal skin points in both treatments over the course of the study. The mean TEWL level of saline treated arms did not differ from that of the Alv treated arms (Fig. 7). Because TEWL measurement could not differentiate between newly healed skin and open skin wounds, this measurement was not valid for evaluation of the wound healing process (Fig. 7).

#### Laboratory data

Several laboratory parameters were measured at d0 (PRE) and d14 (POST) and PRE-POST mean differences were calculated over all patients (Table 4). The values of at least 22 participants were available for each parameter. The mean difference of GO-transaminase indicated a higher mean level on day 14 due to a high difference within d0 and d14 of one participant that was not study related (Mean ± SD = −3.96 ± 22.59; Min = −105, Median = 0). The medians of all mean differences remained at a low level near zero. These results indicated no strong changes between PRE and POST measurements. Paired t-tests were conducted for mean PRE-POST differences and showed no significant results (data not shown). Hence, topical skin treatment of both Alv and control were considered not having any systemic effects.

**Table 3:** Differences in wound healing assessed by %-mean-wound-area.

Visit	Mean Difference	Std Dev	Value of the test statistics	p-value
Day 0	0	0	1.5000	0.500000
Day 2	−5.388730	12.282082	−62.0000	0.037862
Day 4	−7.946144	13.885489	−92.0000	0.002845
Day 6	0	0	.	.
Day 8	0	0	.	.
Day 10	0	0	.	.
Day 12	0	0	.	.
Day 14	0	0	.	.
Day 30	0	0	.	.

An exploratory Wilcoxon signed rank test comparing the %-mean-wound-area between Alv and control treated wounds was significant for d2 and d4. FAS collective, n = 24.

#### Adverse events

Two adverse events occurred during the study (No. 1: cheek abscess, No. 2: patch allergy at the wound dressing attachment site) with a mild grade of severity. Both adverse events were classified as not being related to the study treatment.

## Discussion

By multiple *in vitro* and *in vivo* experimental models as well as a pilot clinical study in human volunteers, we demonstrated the efficacy of lung surfactant in cutaneous healing with anti-inflammatory, pro-migratory and anti-fibrotic effects on skin wound repair.

Lung surfactants are beneficial for wound repair in pulmonary diseases as they influence alveolar surface tension and the inflammatory reaction^27,28^. Commercially available lung surfactant preparations contain the lipophilic fraction after lavage or extraction from animal lungs. Amongst surface emulsifying properties of the phospholipids and the hydrophobic surfactant proteins SP-B and SP-C which ease the absorption and spreading of the surfactant film, phosphatidylglycerol (PG)^29,30^, DPPC^18^, SP-B^31^ and SP-C^32^ have anti-inflammatory and antibacterial^15^ properties^8,14^. Interestingly, surfactant proteins are also secreted by amniotic^33^, skin^34^ and mucosal cells^35^. In scarless fetal wound healing, high amounts of SP-C were found in contrast to almost undectable amounts in adult keratinocytes and fibroblasts. SP-A was predominantly secreted by adult keratinocytes and melanocytes and SP-B and SP-D in all skin cells^34^. As a consequence, the use of lung surfactant to promote skin wound healing and to modulate cutaneous inflammation in order to reduce excessive scarring were addressed in experimental models followed by a human *in vivo* study. First, cell experiments were conducted to study the basic effect of lung surfactant on cellular migration. Next, skin wound repair and inflammation were assessed in a full-thickness animal wound model. This let ultimately to a translational clinical phase I study in healthy volunteers with investigation of the drug’s safety and tolerability when topically applied onto normal, intact skin or superficial wounds.

In contrast to the pro-migratory effect of Alv on keratinocytes in our scratch model which is also found in lung epithelial cells^36^, the behaviour of mesenchymal cells, e.g. fibroblasts, was not influenced by lung surfactant. One can only speculate why high concentrations of 1 mg/mL led to a complete immobilization of both cell types. For example, excess surfactant phospholipids sticking to the cell membrane might have interfered with the extracellular sensing or with the membrane’s surface tension^37^ leading to a migratory stop. Whatever the reasons were, low concentrations of Alv increased epithelial migration and wound closure and were judged as the optimal concentration for further experiments.

Despite of the absence of anti-inflammatory hydrophilic SP-A and SP-D, biological and synthetic lung surfactant preparations or just the phospholipid fraction without any SP can down-regulate the inflammatory response in alveolar monocytic cells^18,29^ and airway epithelial cells^38^. No direct effect of Alv on *TNF* mRNA expression in non-stimulated PBMC was found after 24 h incubation. When PBMC were cultured with conditioned media from scratch-wounded keratinocytes without additives, *TNF* mRNA expression increased significantly, but not in cultures added with 0.01 or 0.5 mg/mL Alv. Obviously, paracrine signalling from injured epithelial cells was down-regulated in presence with Alv with subsequent reduced stimulation of the immune cells’ cytokine production. This contrasts the situation found in the lung with direct effect of surfactant on both epithelial and immune cell derived inflammatory response^38^. Cellular signalling and activation pathways with macrophages being the second phase immune cells arriving at a cutaneous wound site after neutrophils may explain differences seen in both systems^6,7,22^. Another explanation for differences between macrophage activities might be the different cellular origin. Alveolar macrophages derive from fetal monocytic cells and persist in the lung tissue. In contrast, interstitial lung macrophages resemble more their siblings found in skin tissue or PBMC. In fact, alveolar and interstitial lung macrophages show a different pro-inflammatory behaviour with regard to cytokine secretion and express different surface markers^39^. As a consequence, comparisons between skin and lung should focus on interstitial pulmonary macrophages. Furthermore, experimental conditions with monocell cultures exposed to conditioned media of wounded epithelial cells may only partly reflect pathophysiological conditions seen *in vivo*.

Intriguing results yielded *in vivo* experiments using an excisional skin wound model in mice. In parallel to Alv treatment at 0.01 and 0.5 mg/mL, the dissolvent vehicle served as negative control and fatty gauze as reference reflecting the current clinical standard therapy for coverage of acute wounds. No differences with regard to wound closure time were found between the Alv groups and saline treatment, whereas fatty gauze showed fastest reduction of wound area with smallest wound length, but also increased epidermal thickening. Of note, excisional wounds in mice heal primarily by wound contraction in contrast to humans where epithelialization is the main mechanism of wound closure^20^. Macroscopic and histological wound assessment were contrasted by biochemical findings. Increased expression of pro-migratory factors such as *collagenase III (MMP-13)* or *integrin-β6* was found with Alv in contrast to control. During murine skin wound closure, possible beneficial effects of Alv on re-epithelialization were probably masked by wound contraction and, as a consequence, were not detectable by histological analysis.

The cutaneous inflammatory response was significantly reduced with Alv during the observation period. Reduction of pro-inflammatory *TNF, MMP-9, TACE, IL-1β* and *MMP-3* was found in Alv treated wounds in comparison with control. Gene array findings corresponded well with results from qRT-PCR (*TNF*), and immunohistochemistry (CD68). By applying multiple different analytical methods, we demonstrated the anti-inflammatory properties of lung surfactant in skin wounds, similar to the well-known effects on pulmonary epithelia^38^.

Obviously, Alv treatment modulated paracrine signalling of keratinocytes, which attenuated the inflammatory reaction with down-regulation and reduced secretion of TNF by PBMC. This is an important observation, since pro-inflammatory macrophages play a pivotal role in perpetuation of inflammation in chronic venous ulcers^40^ and diabetic wounds^41,42^. By reducing the inflammatory immune cell response, dormant wound repair processes in non-healing wounds could be initiated and excessive scar formation reduced.

In support of this finding, human amniotic membranes release pulmonary surfactant into the amniotic fluid^33^. Accordingly, the use of amniotic membranes has attracted increasing attention to stimulate skin wound healing and enhanced regeneration has been reported of chronic, non-healing^43^ or burn wounds^44,45^ with reduced post-burn scarring^46^. Furthermore, exosomes derived from amniotic epithelial cells stimulated fibroblast migration *in vitro* and skin wound healing *in vivo*^47^. Taken together, all these findings give promising prospects for the future use of lung surfactants to improve skin wound healing and scarring. The reduction of profibrotic *ASMA, CTGF/CCN2, TGF-β2, TGF-βRI, ATII-R2* and *MMP-3* expression by Alv supports even more the potential of lung surfactant as an anti-scarring drug.

Lung surfactant’s pro-migratory and anti-inflammatory effects in preclinical experiments were encouraging to proceed with a translational study to test Alv on human skin *in vivo*. A prospective, randomized clinical phase I study on healthy volunteers was designed with the primary endpoint to test safety and tolerability of the topical application of Alv on intact human skin and on superficial, subepidermal wounds. Lung surfactant proved save and tolerable even though volunteers with atopic background were recruited into the study. Wounds treated with Alv re-epithelialized faster compared to control. This was even more surprising because differences could be seen even though the wounds had a diameter of only 9 mm and were closed within 8 days. This is a novel, hitherto not published observation that shows a direct effect of lung surfactant on skin wound closure in humans *in vivo* and corroborates our *in vitro* results. No drug related adverse events were stated and Alv treatment was equal or superior in all examined parameters. Admittedly, exposure time to lung surfactant was very short with 6 days of treatment in contrast to prolonged treatment periods in patients with chronic wounds. Theoretically, skin sensitization against bovine protein could occur during long-term treatment. Clinically, allergic reactions to topically applied bovine products are extremely rare especially in view of the many wound dressings that contain collagen of bovine origin^48^. Screening literature on allergic reactions to topical exposure to bovine collagen yielded one case report from ophthalmological surgery^49^. Otherwise, various dressings and artificial skin constructs with permanent placement onto wounds showed uneventful healing of chronic wounds^50–53^. In any case, exclusion criteria of our clinical phase I study comprised the item of a known allergy against bovine collagen in order to rule out anaphylactic reactions during treatment.

In agreement with our results with lung surfactant, no graft related adverse events were found with amniotic membrane treatment in diabetic foot ulcers^54^, epidermolysis bullosa^55^ or in burns^56^. Enhancement of granulation tissue formation and wound closure of arteriosclerotic foot sores or amelioration of atopic dermatitis by topical Alv treatment are preliminary observations of a pilot study that will be investigated in the next phase of clinical trials.

In conclusion, lung surfactant and its components have an anti-inflammatory, pro-migratory and anti-fibrotic effect on skin wound healing. Thus, topical application of lung surfactant or its components can have a beneficial effect on human skin wound healing, e.g. acute and chronic wound healing or scarring. By treatment of skin wounds with the lung surfactant Alveofact^®^, wound closure was accelerated significantly and local inflammation attenuated. At the same time, lung surfactant proved safe and tolerable for application onto human skin and, as a consequence, does not impose any safety concerns for clinical use. Our results clearly show that the topical application of lung surfactants is a promising novel and innovative drug-based treatment modality for normal and aberrant human skin wound healing and for prevention for excessive cutaneous scarring.

## Methods

### Ethics

The Ethics Committee of the Medical Chamber of Bremen (no. 336/12 and no. RA/RE 336) *approved* tissue harvest and cell isolation experiments. Skin tissue was obtained from elective plastic surgery and neonatal foreskin fibroblasts from routine circumcision operations after written receipt of patients’ or patients parents’ *informed consent* for tissue donation. With regard to animal experiments, the study was *approved by* the local Ethics Committee of Lower-Saxony (no. 33.9-42502-04-09/1704).

The clinical phase I study was *approved by* the Ethics Committee of the Federal State of Bremen (HB. no. 2015-04-012) and by the Federal Institute for Drugs and Medical Devices (BfArm, no. 4041616). It was registered at EudraCT (no. 2015-000890-11), at the German Clinical Trails Register (DRKS no. DRKS00011353) and at ClinicalTrials.gov (SMWH01), NCT02985437, 7^th^ of December, 2016 (registration date; https://clinicaltrials.gov/ct2/show/NCT02985437). *Informed consent* was obtained from each volunteer prior to the start of the study.

All experiments were carried out *in accordance with* the WMA Declaration of Helsinki (human experiments) or *according to* the guidelines of FELASA (animal experiments). All patients’ data was anonymized so that identifying individual patients was made impossible. Data of the clinical phase I study are securely stored at the Competence Center for Clinical Studies Bremen (University of Bremen) according to German legislation and the current Data protection law (DGSVO).

### Bovine lung surfactant Alveofact^®^

For cell, animal and human studies, the bovine lung surfactant bovactant (Alveofact^®^, Lyomark GmbH, Oberhaching, Germany) was used. Alveofact^®^ (Alv) is extracted from intact bovine lungs by bronchoalveolar lavage. During this process the hydrophilic surfactant proteins (SP) A and D are mostly lost. The highly lipophilic proteins SP-B and SP-C and the surface active lipids are preserved in the extract. According to manufacturer’s information booklet, the mean relative molecular mass of the phospholipids in Alv is 760 Da with a composition of 90% phospholipids, 4% glycerides, 3% cholesterol, 1% SP-B and SP-C, and 0.5% free fatty acids^57^. The phospholipids are composed of 80% phosphatidylcholine, 11% phosphatidylglycerol, 4% phosphatidylethanolamine, 2% sphingomyelin, 1% phosphatidylinositol, 0.6% lysolecithin, 0.5% phosphotidylserine, and 0.2% cardiolipin^57^. The most important phospholipid with regard to surface activity is dipalmitoyl-phosphatidylcholine (DPPC).

Alv lyophilisate was purchased from Lyomark GmbH (experimental studies) and dissolved in the accompanying buffer according to manufacturer’s instructions and then further diluted in 0.9% saline. Immediately after mixing all components, the suspension was added either to cell cultures or topically onto skin wounds at concentrations indicated for each experimental setting. For each time point, fresh Alv solutions were made and used.

### Lung surfactant concentration calculation

Dosage finding studies for cutaneous application of surfactant took into account calculations of the lung surface area and the manufacturer’s recommendations for pulmonal therapy. The alveolar surface (square meters) correlates to the body weight (kg)^58^. To enable calculations, the weight of an average newborn child was put to 2.8 kg with a lung surface area of 2.8 m^2 ^^58^ leading to an alveolar surface area of 1 m^2^/kg. For the acute respiratory distress syndrome in infants, Alv is recommended at a concentration of 50 mg/kg body weight, i.e. 50 mg/m^2^ lung surface. Experimental cell culture experiments were performed in 24 well plates with addition of 1 mL medium onto a surface of 2 cm^2^ per well. Hence the final concentration per well was set to 0.01 mg/mL Alv which corresponded well to the clinically used dosage and which turned out to be also the optimal concentration for *in vitro* and *in vivo* experiments.

### Cell culture experiments

For isolation of primary human keratinocytes and dermal fibroblasts, skin from circumcisions or breast reduction surgery was used, respectively. The fat-free skin was kept at 4 °C in 0.9% saline and used within 4 hours after removal.

#### Keratinocyte and fibroblast isolation

Primary human keratinocytes were obtained from foreskin tissue of boys less than one year of age, while primary human fibroblasts were harvested from excised skin tissue after plastic surgery. The tissues were stored in Hank’s solution with antibiotics (200 IU/mL penicillin, 200 µg/mL streptomycin). Afterwards tissues were minced, treated with 0.1% collagenase (Serva, Heidelberg, Germany) and incubated for 3 h to 4 h at 37 °C to separate the epidermal and dermal layer. Keratinocytes were gained from the epidermis, fibroblasts were taken from the dermal layer. The cells were cultured using EpiLife medium (Thermo Fisher Scientific, Schwerte, Germany) for keratinocytes or TC199 medium with 20% FCS for fibroblasts at 37 °C in 5% CO_2_ air. The culture medium was changed after attachment of the cells. The cells were passaged using trypsin/EDTA solution (0.05%/0.02% w/v in PBS without Ca^2+^, Biochrom, Berlin, Germany) in a split ratio of 1:2 once a week to preserve monolayer formation.

#### Transmission electron microscopy imaging for intracellular Alv detection

Control (saline) or Alv treated cells were fixed in 3% glutardialdehyde at 4 °C and further processed as described previously^59^ and analysed using a transmission electron microscope (Margagni 268, FEI, Eindhoven, Netherlands).

#### Human PBMC culture with keratinocyte-conditioned medium

Human peripheral blood mononuclear cells (PBMCs) were isolated from blood buffy coats of anonymous healthy donors (Blutspendedienst Hamburg, Hamburg, Germany) using Ficoll-Paque Plus (GE Healthcare, Chicago, IL) gradient as described before^60^. In parallel, keratinocytes treated without or with 0.01 mg/mL or 0.5 mg/mL Alv for 24 h and conditioned media were collected for addition to PBMC cultures. Then PBMCs were cultured for 0 h (control) or 4 h with keratinocyte conditioned medium followed by collection of cell pellets for *TNF* mRNA expression analyses and supernatant collection for TNF ELISA analysis. As positive control for *TNF* expression, 10 ng/mL LPS were used.

#### Scratch tests of keratinocyte or fibroblast monocultures

Scratch wounding of cell cultures was performed as described previously^61^. In brief, cell monocultures were grown to 100% confluence. With a 200 µL pipette tip a scratch was made in the middle of each well resembling an *in vivo* wound. Cell migration was captured after 24 and 48 h using an Olympus VK microscope at a magnification of 4×. Pictures were digitized and analysed with ImageJ™. Keratinocyte motility was captured by video imaging for 24 hours. Phase contrast images were acquired every two minutes using an Axiovert 135 TV epifluorescence microscope connected to a DCMC-800 camera. During the experiment cells were enclosed in a home-built chamber, controlling 37 °C temperature and 5% CO_2_ concentration. *Fiji software*^62^ was used to measure the area of the gap during the time as previously reported^63,64^. The ratio *A/A*_0_ was plotted versus the observation time, where *A*_0_ is the initial area of the scratch and *A* is the area at different times. Scratch assays were repeated in triplicate with primary keratinocytes from different donors testing concentrations of 0.01 and 0.1 mg/mL Alv in comparison to controls (plain medium).

#### Fibroblast contraction assay in free-floating collagen lattices

Free-floating collagen lattices were prepared as described elsewhere^65^. In short, human primary dermal fibroblasts from three different donors were used between passages 4 and 6 and incorporated into collagen gels at a concentration of 10^5^cells/ml. After 30 min stacking time, media (DMEM + 10% FCS) with or without treatments were added to each well. Gels were cultured under standard conditions and contraction monitored over 120 h. Gel contraction was monitored using an Olympus SX microscope, digitized and analyzed using ImageJ™ software.

### Excisional full-thickness skin wound model in mice

The effect of Alv on skin wound healing was assessed *in vivo* using a standard experimental model creating four full-thickness excisional wounds on the back of Black-6 mice (male, 8 weeks of age, C57BL/6NCrl; Charles River) with an 8 mm trephine^66^. Each mouse received one single treatment onto wounds, e.g. Alv was applied onto wounds at 0.01 mg/mL or 0.5 mg/mL. Saline served as negative control, fatty gauze as clinical standard treatment control. Wound dressings were changed every second day with new application of treatments. After treatment substance application, wounds were covered with sterile gauze, followed by dressing fixation using Lomir mouse jackets, (Lomir Biomedical Inc., Malone, NY). Animals were sacrificed after 8 or 14 days and tissues harvested for further analyses. Wound margins were traced and wound areas analysed as described elsewhere^66^. All experimental analyses were performed in a blinded manner.

### Histology and immunohistochemistry

Tissues were fixed in 4% PBS buffered PFA and then processed as described elsewhere^67^. Wound width and epidermal thickness were measured in hematoxylin-eosin (HE)-stained sections using an Olympus SX microscope. Data were analysed with ImageJ™ software. Myofibroblast or macrophage occurrence and vessel formation were addressed by immunohistochemistry for α-smooth muscle actin (ASMA), CD68 or collagen type IV, respectively. Paraffin sections were stained using primary and secondary antibodies listed in Suppl. Table 1. Immunostaining was performed by the avidin-biotin-peroxidase complex technique (PK-4000, Vectastain ABC Kit; Vector Laboratories, Burlingame, CA). Diaminobenzidine was used as a chromogenic substrate and hematoxylin as counterstain as described in detail elsewhere^68^.

Ki67 positive epithelial cells were counted within an epidermal length of 600 µm of the wound margin and 500 µm of the wound centre and expressed as number of cells per 100 µm. Caspase-14 and ASMA positivity were semi-quantitatively analysed by two independent researchers in a blinded way for each wound and intensities ranked from 0 to 3, e.g. 0 no staining, 1 weak, 2 moderate, and 3 abundant staining. Inflammatory cell infiltration in wound granulation tissue was analysed by CD68 immunohistochemistry for macrophages at a magnification of 10×. Sections were scanned and digitized, the whole area of the section calculated in mm^2^ using NIS-Elements microscope imaging software (Nikon Metrology NV). CD68 positive cells were counted over the entire histological slide and stated as positive cells per area. All immunohistochemical assessments were performed in a blinded way.

### Biochemical analyses

Biochemical analyses were performed as described previously, e.g. qRT-PCR^69,70^ and gene array analysis^71^. For ELISA analyses, skin was homogenized as described elsewhere^23^. Protein content was quantified using the BCA method (Pierce, Rockford, IL, USA). TNF was measured in conditioned media and tissue homogenates using the human TNF-α DuoSet ELISA (DY210-05, R&D Systems) according to manufacturer’s instructions.

#### RNA extraction

Wound samples were frozen in liquid nitrogen and stored at −80 °C. Samples were kept deep frozen in liquid nitrogen and cut into small pieces about 1 mm². Pieces were then left in 1 ml TRIzol® Reagent (Life Technologies; #15596-026) for 5 min at 20 °C after being homogenised with a Polytron PT1200E. RNA isolation was performed according BioRad/Bioscience technology (MIQE Guidelines). The resulting RNA pellet was resuspended in 20 µl DMPC water and stored at −20 °C. Obtained RNA was quantified with a NanoDrop 1000 and its quality was appreciated through an agarose gel electrophoresis by observation of the 28S and 18S bands.

#### Gene expression analysis by tailor-made array

A new Wound Tissue microarray containing 164 genes of *Mus musculus* involved in skin wound healing, inflammation and scarring was developed for this study. Subgroups of analysed genes according to family or function are stated in Fig. 2 (for nomenclature see Suppl. Table 2). The complete list of arrayed genes is available online at the GEO record GPL24597 (http://www.ncbi.nlm.nih.gov/geo/). The 50mer oligonucleotides were designed and purchased from Invitrogen (Darmstadt, Germany) and spotted on aldehyde modified glass slides (VSS25, CEL Associates, Inc., Pearland, TX, USA) using an Affymetrix 417 arrayer. Oligonucleotide solutions were prepared in 96-well-plates containing each 30 µl. The concentration of the printed oligonucleotides was 50 µM in 1x ArrayIt^TM^ Microarray Spotting Solution Plus (CEL Associates, Inc., Pearland, TX, USA). After spotting, the slides were incubated at 80 °C for 2 h. Slides were blocked in SSC containing 1% BSA (Sigma) for 45 min and rinsed with water for 5 min.

##### cDNA synthesis and purification

Total RNA was isolated using the Aurum total fatty and fibrous tissue kit (Biorad, Munich, Germany). For cDNA synthesis the *NEN*^*®*^ *Micromax TSA Labeling and Detection Kit* (PerkinElmer, Rodgau-Jügesheim, Germany) was used. During reverse transcription 6 µg of purified total RNA ISC was converted into fluorescein (Fl) and biotin (B) labelled cDNA. Purification of the fluorescein and biotin labelled cDNA was performed using the *QIAquick PCR Purification Kit* according to the manufacturer’s instructions (Qiagen, Hilden, Germany). Additionally, cDNA was purified with 35% guanidine hydrochloride solution and eluted twice with *EB buffer* (1:10 dilution, pH 8.5).

##### Hybridisation

Hybridisation was performed in a special form of a dye-swap, a so called loop design^72^. Loop design means a hybridisation procedure with a combination of different labelled cDNA targets that minimizes the number of the required chips. The transcriptional effects of the three different surface substrates were directly compared resulting in three differently labelled chips. Thus, the purified fluorescein and biotin labelled cDNA of two samples were hybridised simultaneously in one experiment to the same array over night at 42 °C in a humidified surrounding.

##### Washing and detection

After hybridisation, non-specifically bound cDNA was removed by stringent washing from the array. Specifically bound fluorescein and biotin labelled cDNAs were sequentially detected with a series of conjugate reporter molecules according to the TSA process, ultimately with tyramide-Cy3 and tyramide-Cy5, as described elsewhere^73,74^.

##### Scanning

Subsequently the hybridised array was scanned for the two distinct fluorescent dyes (Cy3 and Cy5) of the cDNA derived from the two differently treated cells with an Axon 4000 B (Axon Instruments, Inc., Union City, CA) confocal array scanner. The chip was scanned at six different settings changing PMT or laser power.

Primary and secondary data analysis. A microarray analysis program addressing the individual issues has been written in Matlab R2006b^75^. Primary data from each array were collected with GenePix 6.0 software. Therefore, the arrays were scanned different times with a 4000 B scanner. Images were quantified using GenePixPro 6.0 software and analysed as reported previously^75–77^. The gene replicates were tested for outliers with the Nalimov test and the remaining data for each gene were averaged. Genes were grouped according to gene family or biological function. The whole dataset can be accessed via the Series GSE110438 in the Gene Expression Omnibus database (GEO, http://www.ncbi.nlm.nih.gov/geo/). Values are stated in Fig. 2, the nomenclature of values is provided in Suppl. Table 2. The clustergram graphics were obtained using the clustergram function from Matlab 2017b^78^. The complete data set is shown in Suppl. Fig. 2 whereas detailed excerpts of these data are provided in Figs. 3–5 and Suppl. Fig. 1.

#### TNF RNA expression in mouse skin and human PBMCs

For mouse skin, total RNA was isolated as stated above. For human PBMCs, total RNA was isolated from PBMC pellets with a Trizol extraction system (TriFast, PEQLAB GmbH, Erlangen, Germany), cDNA synthesis and quantitative RT-PCR was performed as previously described^69,70^. The Applied Biosystems StepOne Real-Time PCR system (Applied Biosystems, Forster City, CA) and TaqMan® Fast Universal PCR Master Mix for TaqMan assays (Applied Biosystems) were used for analysis. β-actin and cyclophilin were used as internal housekeeping controls and the quantitative analysis was performed with the ΔΔCT method. The following TaqMan® Gene Expression Assays (Applied Biosystems) were used: mouse *Actb* (Mm00607939_s1), mouse *Tnf* (Mm00443258_m1), human *TNF* (Hs99999043_m1), human *PPIA* (Hs99999904_m1).

All pre-clinical experimental analyses were performed in a blinded manner.

### Clinical phase I study

#### Determination of sample size

The study was powered to show non inferiority in the primary end point. The null hypothesis to be tested was non inferiority of Surfactant versus NaCl in the mean CSS with an NI margin of 1 score point. For the sample size calculation using the t-test the variance was assumed to be equal to 1 score point. The one-sided significance level employed was 0.025. Calculations using nQuery Advisor revealed that in total 23 subjects were needed to achieve a power of 90% if the true difference between CSS means is equal to zero (equal tolerability of Surfactant and NaCl treatment). Since the randomization was stratified by sex an even number of subjects was needed. Therefore, the required sample size for this trial was calculated to be 24.

This sample size calculation was further supported by Monte Carlo simulations using the Wilcoxon signed rank test. These simulations indicated that 24 samples were sufficient to detect differences between Surfactant and NaCl for combinations of an alpha of around 5% and betas between 10% and 25%, depending on the model used for the generation of the scores.

#### Randomization of study participants

To avoid a systemic error, a randomization stratified by sex was performed by the Competence Center for Clinical Studies Bremen, University of Bremen, on which lower arm the control or the Surfactant substance was to be applied. Randomized allocation of treatment arms were stated in a sealed envelope that was broken for each participant immediately after start of the study with wounding. Applying both treatments to all individuals had the advantage that each individual could serve as its own control, leading to homogenous treatment groups and a smaller sample size. No blinding was performed since it was not regarded necessary because this is a study for safety assessment. Besides, the treatment fluid containing lung Surfactant has a milky appearance whereas the control vehicle, 0.9% sodium chloride solution, is a clear fluid making blinding hard to accomplish.

#### Study design

Twenty-four healthy volunteers were enrolled into this prospective, randomized non-blinded clinical phase I study which took place at the University of Bremen, Bremen. For further details and eligibility criteria, please visit https://clinicaltrials.gov/ct2/show/NCT02985437. Safety and tolerability of Alveofact^®^ was assessed on normal, non-injured forearm skin and on subepidermal suction blister wounds. After creation of four 9-mm suction blisters on each forearm^24^ blister roofs were excised aseptically and treated either with 0.5 mg/mL Alv (randomised: one arm) or saline alone (control: the other arm). A semi-occlusive dressing was applied on top of each treatment with OPSITE POST-OP^®^ dressing (#1447447, smith&nephew, UK). Dressings were exchanged every other day until wound closure. For the primary target variable, normal skin and wounds were assessed by a clinician using a clinical score scale (CSS)^79^. Pictures of normal skin and wounds were taken, digitised and wound areas measured using ImageJ™^80^. For the secondary target variables, pain was scored using the numerical rating scale (NRS, commonly called visual analogue scale)^81^ and epithelial resurfacing was assessed by measurement of wound areas and by measurement of trans-epidermal water loss (TEWL)^24^ above wounds. TEWL was assessed on four wound points placed clockwise starting with W1 at 3 o’clock (W1–4), on adjacent healthy skin (N3–4; covered by dressing) and on uncovered healthy skin points (N1–2; Fig. 7). The equipment used was the Tewameter TM300 (Courage + Khazaka electronic GmbH, Cologne, Germany) at ambient temperature of 20–22 °C and relative humidity of 30–40%^24^. Blood samples were taken before blister formation on d0 and on postoperative d14. The observation period extended over 30 days. Visits were scheduled every other day until d14. At the end of the observation period (day 30) a follow-up visit was held.

### Statistics

#### Statistics experimental analyses

Normal distribution of data was analyzed using the Shapiro-Wilk-Test. If not otherwise stated, the Student’s t-test was used for data with normal distribution and the Whitney-Mann-U-test otherwise (Graphpad Prism™ 8.0.2 software, San Diego, CA). Values of p < 0.05 were assumed as significant and expressed as Mean ± SD (standard deviation) or SEM (standard error of the mean). For cell migration analysis and ASMA expression, the one-way ANOVA test was used and results corrected after Bonferroni for multiple comparisons. For analysis of gene array results, the averaged values for each gene comprised nine replicates. Outliers amongst the gene replicates were eliminated according to the outlier test by Nalimov. An adaption of Student’s t-test, e.g. Welch’s t-test for unequal variances of the analysed samples was used for comparison of means.

#### Statistics clinical phase I study

The analysis of the primary and secondary target variables was performed on the per-protocol set (PP) or the full-analysis set (FAS) (see also flow chart diagram Fig. 6a). For analysis of the CSS and NRS, both acting as safety parameters, the PP-Set was used. Missing interim data for the CSS and NRS (after wound closure) was imputed using linear imputation. If all CSS/NRS data after a certain visit were missing they were imputed using the LOCF (last observation carried forward) method. For each patient the mean CSS/NRS score (over all visits) was calculated per arm. The difference in means of CSS/NRS (over all visits) between both arms was tested with a one-sided Wilcoxon signed rank test for non-inferiority of Alv compared to saline on a significance level of 0.025. The non-inferiority margin employed was 1 score point for CSS as well as NRS.

For analysis of the TEWL, the FAS was used. Missing interim data was imputed using linear interpolation. Subsequent missing values after drop out/withdrawal were imputed using the LOCF method. For each patient and each visit 15 repeated measurements per measurement point were available. Aggregated mean values for healthy skin (N1, N2), adjacent healthy skin (N3, N4) and wounds (W1, W2, W3, W4) were calculated per arm and each visit (see Fig. 7A for definition of measurement points). For each visit a two-sided paired t-test comparing these mean TEWL values between Alv treated arms and control treated arms was performed.

For wound area analysis (post-hoc) the mean wound area (in mm² over all patients for available values) per arm was calculated relative to baseline (just after building lesions, i.e. baseline: 100%, complete wound closure: 0%). For each visit, a two-sided Wilcoxon signed rank test comparing the %-mean-wound-area of the Alv treated arms to that of the control treated arms was performed. Since all tests done for the secondary target variables were of exploratory nature only, no adjustment for multiplicity was done. Sensitivity analyses using the analysis set not used for the main analysis were performed and supported the main analysis in every case.

### Trial registration

The clinical phase I study was registered at EudraCT (no. 2015-000890-11), at the German Clinical Trails Register (DRKS no. DRKS00011353) and at ClinicalTrials.gov (SMWH01). The summary of test statistics of metric baseline variables are shown in Table 1. The complete data set of laboratory results are shown in Table 4.

**Table 4:** Laboratory data.

Variable	N	Mean ± SD	Min	Q1	Median	Q3	Max		
**Differences in laboratory parameters between day 0 and day 14**									
CRP (mg/l)	23	0.73 ± 4.55	−5.50	−1.10	0.00	0.80	18.60		
Creatinine (mg/dl)	23	0.02 ± 0.07	−0.11	−0.03	0.02	0.05	0.24		
Erythrocytes (1/pl)	23	0.03 ± 0.26	−0.50	−0.20	0.00	0.20	0.50		
Glucose (mg/dl)	23	−0.48 ± 18.66	−32.00	−11.00	−4.00	12.00	42.00		
GOT (U/l)	23	−3.96 ± 22.59	−105.00	−4.00	0.00	5.00	9.00		
GPT (U/l)	22	−1.00 ± 6.34	−19.00	−3.00	0.50	4.00	6.00		
Hemoglobin (g/dl)	23	0.08 ± 0.79	−1.50	−0.40	0.00	0.80	1.40		
Leukocytes (1/nl)	23	−0.10 ± 1.57	−2.85	−1.30	0.00	1.51	2.30		
Potassium (mmol/l)	22	0.03 ± 0.29	−0.40	−0.20	0.05	0.20	0.70		
PPT (s)	22	−0.04 ± 1.08	−3.90	−0.40	0.05	0.70	1.30		
PT (%)	23	2.48 ± 6.56	−13.10	−1.00	2.00	7.30	13.80		
Sodium (mmol/l)	23	1.09 ± 2.91	−4.00	−1.00	1.00	1.00	8.00		
Thrombocytes (1/nl)	23	3.48 ± 37.23	−70.00	−20.00	5.00	20.00	99.00		
Urea (mg/dl)	23	0.80 ± 7.09	−10.00	−5.10	0.00	6.30	15.80		
**Laboratory parameters before start of the study on day 0**									
	**N**	**Missing**	**Mean**	**Std Dev**	**Min**	**Q1**	**Median**	**Q3**	**Max**
CRP (mg/l)	23	1	3.18	5.42	0.30	0.30	0.60	3.18	5.42
Creatinine (mg/dl)	23	1	0.65	0.13	0.43	0.57	0.66	0.65	0.13
Erythrocytes (1/pl)	23	1	4.71	0.50	3.80	4.40	4.60	4.71	0.50
Glucose (mg/dl)	23	1	91.83	17.42	66.00	76.00	88.00	91.83	17.42
GOT (U/l)	23	1	28.52	6.87	21.00	24.00	27.00	28.52	6.87
GPT (U/l)	23	1	23.13	15.04	10.00	15.00	20.00	23.13	15.04
Hemoglobin (g/dl)	23	1	13.90	1.66	10.90	12.50	13.50	13.90	1.66
Leukocytes (1/nl)	23	1	7.24	1.56	5.12	6.30	6.72	7.24	1.56
Potassium (mmol/l)	23	1	4.56	0.33	4.00	4.30	4.60	4.56	0.33
PPT (s)	22	2	28.75	2.74	22.40	27.80	28.60	28.75	2.74
PT (%)	23	1	96.05	10.28	75.10	90.30	98.40	96.05	10.28
Sodium (mmol/l)	23	1	140.35	2.85	134.00	138.00	140.00	140.35	2.85
Thrombocytes (1/nl)	23	1	274.43	78.74	130.00	215.00	275.00	274.43	78.74
Urea (mg/dl)	23	1	23.51	7.00	13.40	18.30	22.40	23.51	7.00
**Laboratory parameters on day 14**									
	**N**	**Missing**	**Mean**	**Std Dev**	**Min**	**Q1**	**Median**	**Q3**	**Max**
CRP (mg/l)	24	0	2.36	3.71	0.30	0.30	0.65	2.45	15.60
Creatinine (mg/dl)	24	0	0.64	0.11	0.44	0.56	0.64	0.72	0.85
Erythrocytes (1/pl)	24	0	4.71	0.44	4.00	4.45	4.70	4.95	5.50
Glucose (mg/dl)	24	0	91.25	11.10	67.00	86.50	92.50	98.50	111.00
GOT (U/l)	24	0	32.63	24.54	18.00	23.50	27.00	31.50	141.00*
GPT (U/l)	23	1	24.35	19.15	9.00	16.00	18.00	24.00	103.00
Hemoglobin (g/dl)	24	0	13.89	1.50	11.30	13.00	13.75	15.10	16.60
Leukocytes (1/nl)	24	0	7.39	2.01	4.33	6.08	7.62	7.92	14.28
Potassium (mmol/l)	23	1	4.55	0.35	4.10	4.20	4.50	4.90	5.20
PPT (s)	24	0	29.02	2.98	22.40	27.35	28.90	29.90	35.60
PT (%)	24	0	93.96	9.57	74.30	88.50	91.35	102.75	111.90
Sodium (mmol/l)	24	0	139.33	2.12	136.00	138.00	139.50	141.00	143.00
Thrombocytes (1/nl)	24	0	267.50	80.96	139.00	204.00	273.50	308.00	464.00
Urea (mg/dl)	24	0	22.85	6.26	10.90	19.25	22.85	27.30	33.80

*High maximal GOT due to an outlier value of one participant at day 14 that was not study related.

## Supplementary information

Dataset 1.

Suppl Video 1.

Suppl Video 2.

Suppl Video 3.

## Data Availability

**Gene expression analysis by tailor-made array**. The complete list of arrayed genes is available online at the GEO record GPL24597 (http://www.ncbi.nlm.nih.gov/geo/). The complete data of the gene array results are listed in Fig. 2a,b. The nomenclature of genes is listed in Supplementary Table 2. Clustergrams of analyzed genes are shown in Figs. 3–5 and Supplementary Figs. 1 and 2.
