# Supplementary material for: Lung Surfactant Accelerates Skin Wound Healing: A Translational Study with a Randomized Clinical Phase I Study
Source: Sci Rep. 2020 Feb 13;10:2581. doi: 10.1038/s41598-020-59394-5 (PMC7018835; doi:10.1038/s41598-020-59394-5)
Supplement: Supplementary file 1 — Dataset 1. [file 41598_2020_59394_MOESM1_ESM.pdf]

LUNG SURFACTANT ACCELERATES SKIN WOUND HEALING: A TRANSLATIONAL STUDY WITH A RANDOMIZED CLINICAL PHASE I STUDY

Ursula Mirastschijski[1,*;†]; Igor Schwab,[2,†] Vincent Coger,[3] Ulrich Zier,[1] Carmela Rianna[4], Wei He[1], Kathrin Maedler[1], Sørge Kelm[1], Arlo Radtke[1], Gazanfer Belge[1], Patrick Lindner,[5] Frank Stahl[5], Martin Scharpenberg[6], Lukas Lasota,[6] Jürgen Timm[6]

[1]Center for Biomolecular Interactions Bremen, Faculty of Biology and Chemistry, University of Bremen, Bremen, Germany;

[2]Department of Plastic, Reconstructive and Aesthetic Surgery, Klinikum Bremen-Mitte, Bremen;

[3]Department of Experimental Plastic Surgery, Kerstin Reimers Laboratory for Regeneration Biology, Hannover Medical School, Hannover, Germany;

[4]Institute of Biophysics, University of Bremen;

[5]Institute of Technical Chemistry, Leibniz University Hannover, Hannover;

[6]University of Bremen, Competence Center for Clinical Trials Bremen, Bremen.

†authors contributed equally.

SUPPLEMENTARY FIGURES

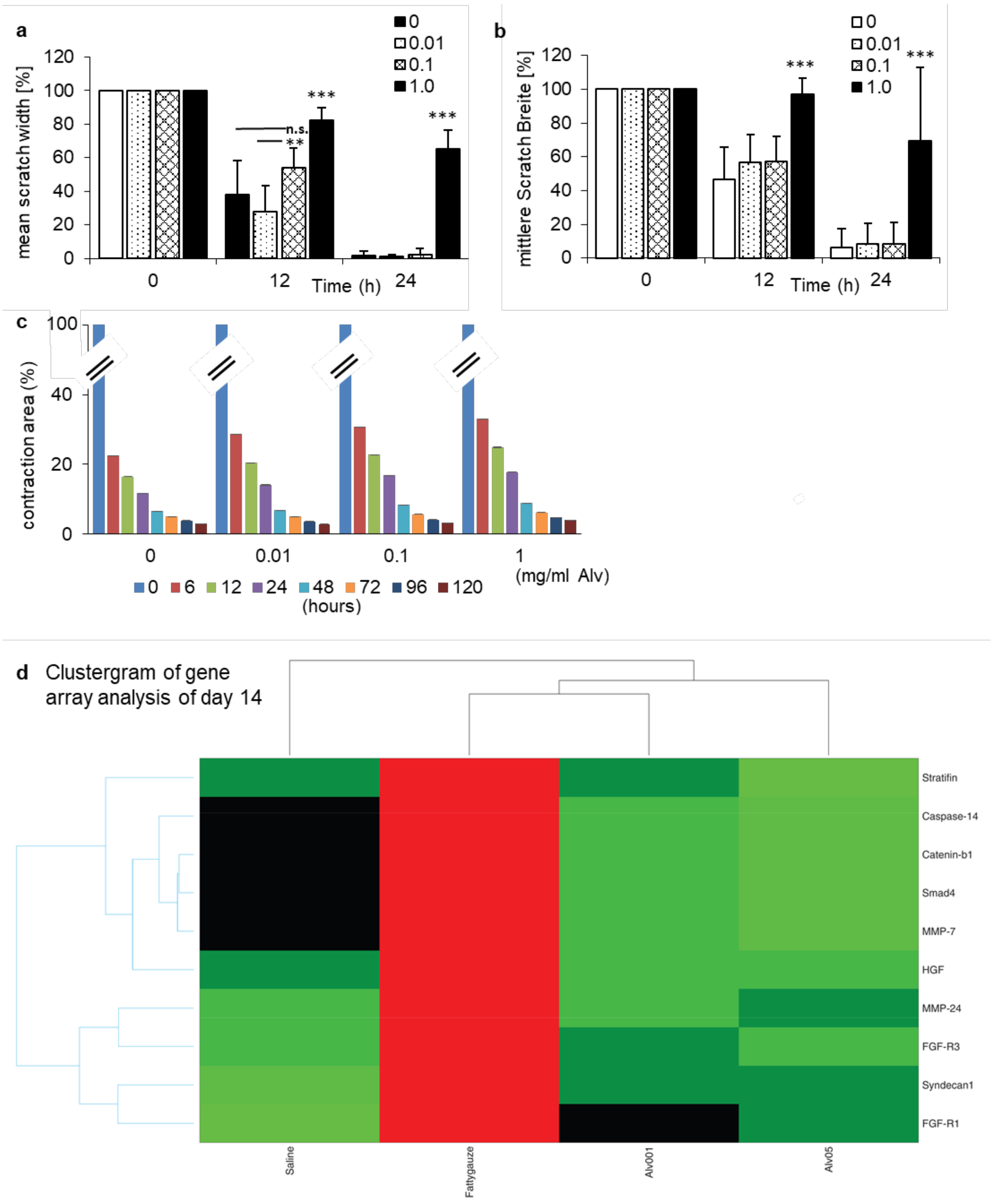

Suppl. Figure 1. Effect of Alv on keratinocyte and fibroblast migration and contraction. Keratinocyte (a) or fibroblast (b) migration with or without Alv at different concentrations. a. Alv at 0.01 mg/mL increased keratinocyte migration over a scratch compared to control, whereas fibroblast migration was unchanged. At concentration of 1 mg/mL, cellular migration was severely and statistically significantly (****p*<0.001) inhibited. White bars control (saline), dotted bars Alv at 0.01, hatched bars Alv at 0.1 and black bars Alv at 1.0 mg/mL.

n=9. Mean ± SEM. (ANOVA test, Bonferroni correction) c. Fibroblast contraction of free-floating collagen lattices was not affected by Alv over the 120h observation period. d. Caspase-14 was reduced with Alv 0.01 and 0.5 compared to control and an increase with fatty gauze (FG) by gene array analysis on d14.

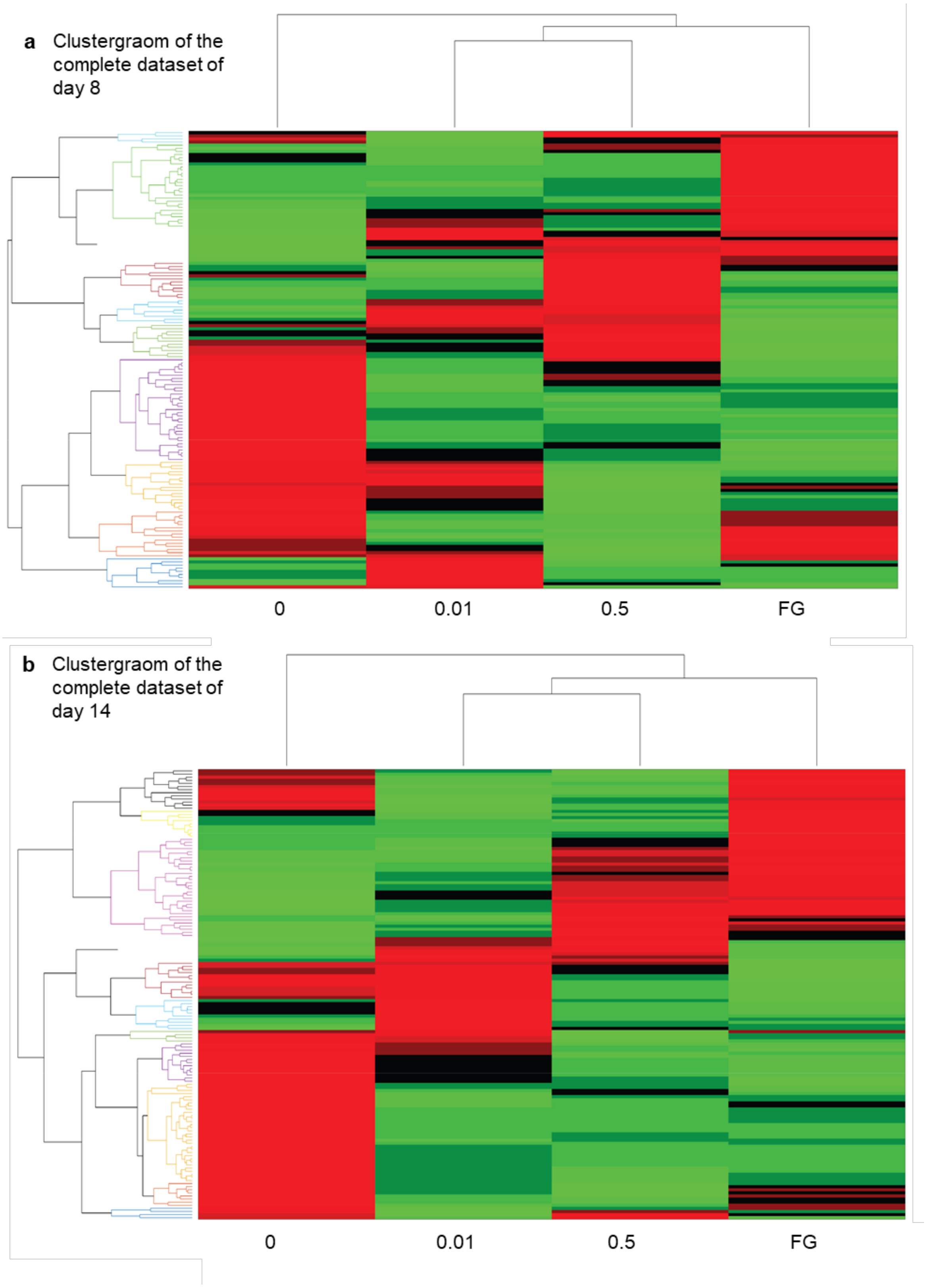

Suppl. Figure 2. Clustergram of gene array showing up- and downregulation of all analyzed genes in comparison to control. a. Gene regulation on day 8. b. Gene regulation on day 14. Data are presented with a standard red-green-map in which red represents values above the mean, black represents the mean, and green represents values below the mean of a row (gene) across all columns (samples). More detailed information is provided in Fig. 2-5, and Suppl. Table 2. 0 control, FG fatty gauze, Alv at 0.01 and 0.5 mg/mL.

SUPPLEMENTARY TABLES

Suppl. Table 1. Antibodies used for immunohistochemisty

	Dilution	Catalogue no	Company	Sec. antibody
Ki67	1:300	RM-9106-S0	Thermo-Scientific	E432, DAKO
Caspase-14	1:300	IMB-5713	Imgenex	E432, DAKO
ASMA	1:300	Ab5694	Abcam	E432, DAKO
Collagen IV	1:2000	Ab6586	Abcam	E432, DAKO
CD68	1:100	GTX37743	GeneTex	E432, DAKO

Suppl. Table 2. Nomenclature of genes measured by the gene array analysis

**Abbreviation**	**Full description of the gene**
ASMA	Alpha smooth muscle actin
ATII-R1A	Angiotensin II-Receptor 1A
ATII-R1B	Angiotensin II-Receptor 1B
ATII-R2	Angiotensin II-Receptor 2
ATogen	Angiotensionogen
BetaActin	β-actin
Caldesmon1	Caldesmon-1
Caspase14	Caspase-14
Caspase3	Caspase-3
Caspase8	Caspase-8
Catenin-a2	α2-Catenin
Catenin-b1	β1-Catenin
CathepsinK	Cathepsin-K
CathepsinL	Cathepsin-L
CathepsinS	Cathepsin-S
Claudin6	Claudin-6
Cofilin	Cofilin
Connexin26	Connexin-26

Connexin30	Connexin-30
Connexin31	Connexin-31
Connexin311	Connexin-311
Connexin43	Connexin-43
CTGF	Connective tissue growth factor/CCN2
Decorin	Decorin
Desmocollin1	Desmocollin-1
Desmocollin2	Desmocollin-2
Desmocollin3	Desmocollin-3
Desmoglein1	Desmoglein-1
Desmoglein3	Desmoglein-3
Desmoglein4	Desmoglein-4
Desmoplakin III	Desmoplakin III
ECadherin	E-Cadherin
EGF	Epithelial growth factor
EGFR	Epithelial growth factor Receptor
Elastin	Elastin
Endothelin I	Endothelin I
FGF1	Fibroblast growth factor-1
FGF2	Fibroblast growth factor-2
FGF9	Fibroblast growth factor-9
FGF-R1	Fibroblast growth factor-Receptor 1
FGF-R2	Fibroblast growth factor-Receptor 2
FGF-R3	Fibroblast growth factor-Receptor 3
Fibromodulin	Fibromodulin
Fibronectin	Fibronectin
Filaggrin	Filaggrin
Follistatin	Follistatin
Glypican1	Glypican-1
Glypican3	Glypican-3
HB-EGF	Heparin binding- Epithelial growth factor
HGF	Hepatocyte growth factor
IGF1	Insullin-like growth factor-1
IL10	Interleukin-10
IL1b	Interleukin-1β
IL6	Interleukin-6
Integrin-a5	Integrin-α 5
Integrin-a6	Intergrin-α 6
Integrin-aE	Integrin-α E
Integrin-aV	Integrin-α V
Integrin-b1	Integrin-β1
Integrin-b3	Integrin-β3
Integrin-b4	Integrin-β4
Integrin-b5	Integrin-β5
Integrin-b6	Integrin-β6
Integrin-b7	Integrin-β7
Integrin-b8	Integrin-β8

Involucrin	Involucrin
Keratin14	Keratin-14
Keratin5	Keratin-5
Ki67	Ki67
Collagen Ia1	Collagen-I α a1
Collagen Ia2	Collagen-I α a2
Collagen IIIa1	Collagen-III α a1
Collagen IVa1	Collagen-IV α a1
Collagen IVa6	Collagen-IV α a6
Collagen Va1	Collagen-V α a1
Collagen Va2	Collagen-V α a2
Collagen VII	Collagen-VII
Collagen XVII	Collagen-XVII
Laminin-a3	Laminin-α3
Laminin-b1	Laminin-β1
Laminin-b3	Laminin-β3
Laminin-g1	Laminin-γ1
Laminin-g2	Laminin-γ2
LOX	Lysyl oxigenase
LTGFbBP	Latent TGF-β-binding protein
MMP-1a	Matrix metalloproteinase-1a
MMP-1b	Matrix metalloproteinase-1b
MMP-2	Matrix metalloproteinase-2
MMP-3	Matrix metalloproteinase-3
MMP-7	Matrix metalloproteinase-7
MMP-8	Matrix metalloproteinase-8
MMP-9	Matrix metalloproteinase-9
MMP-12	Matrix metalloproteinase-12
MMP-13	Matrix metalloproteinase-13
MMP-14	Matrix metalloproteinase-14
MMP-15	Matrix metalloproteinase-15
MMP-21	Matrix metalloproteinase-21
MMP-24	Matrix metalloproteinase-24
MMP-27	Matrix metalloproteinase-27
MMP-28	Matrix metalloproteinase-28
NCadherin	N-Cadherin
Nidogen1	Nidogen-1
OBCadherin	OB-Cadherin
Occludin	Occludin
PDGFA	Platelet derived growth factor
PDGFAR	Platelet derived growth factor-Receptor A
Plasminogen	Plasminogen
Plectin1	Plectin-1
Plod1	Procollagen-lysine, 2-oxoglutarate 5-dioxygenase-1
Plod3	Procollagen-lysine, 2-oxoglutarate 5-dioxygenase-3
Seprase	Seprase
Serpin-e1	Serpin-e1

Serpin-eh1	Serpin-e human1
SLPI	Secretory leukocyte protease inhibitor
Smad2	Smad2
Smad3	Smad3
Smad4	Smad4
Smad6	Smad6
Smad7	Smad7
Stratifin	Stratifin
Syndecan1	Syndecan-1
TACE	TNF-α converting enzyme
Tenascinc	Tenascin-C
Tensin1	Tensin-1
Tensin2	Tensin-2
Tensin3	Tensin-3
Tensin4	Tensin-4
TGFa	Transforming growth factor-α
TGFb1	Transforming growth factor-β1
TGFb2	Transforming growth factor-β2
TGFb3	Transforming growth factor-β3
TGFb-RI	Transforming growth factor-β Receptor I
TGFb-RII	Transforming growth factor-β Receptor II
TGFb-RIII	Transforming growth factor-β Receptor III
TIMP1	Tissue Inhibitor of Matrix metalloproteinases-1
TIMP2	Tissue Inhibitor of Matrix metalloproteinases-2
TIMP3	Tissue Inhibitor of Matrix metalloproteinases-3
TLR6	Toll-like Receptor 6
TLR7	Toll-like Receptor 7
TLR8	Toll-like Receptor 8
TNF	Tumor necrosis factor
TNFa-R1	Tumor necrosis factor-α Receptor 1
TNFa-R2	Tumor necrosis factor-α Receptor 2
uPA	Urokinase Plasminogen activator
VEGFA	Vascular endothelial growth factor-α
Versican	Versican
Vinculin	Vinculin
Vitronectin	Vitronectin

SUPPLEMENTARY VIDEOS

Suppl. Video 1-3. Videos show scratch tests on untreated keratinocytes (video1) and keratinocytes treated with 0.01 and 0.1 mg/mL Alv (video2 and 3, respectively). Tests were performed over 24 hours. The migratory behaviour of primary keratinocytes varied depending on their origin from different donors. Video3 has different quality because of a technical problem with the camera.
